# Saponins as Natural Emulsifiers for Nanoemulsions

**DOI:** 10.1021/acs.jafc.1c07893

**Published:** 2022-05-27

**Authors:** Tatiana B. Schreiner, Madalena M. Dias, Maria Filomena Barreiro, Simão P. Pinho

**Affiliations:** † Centro de Investigação de Montanha (CIMO), Instituto Politécnico de Bragança, Campus de Santa Apolónia, 5300-253 Bragança, Portugal; ‡ LSRE-LCM - Laboratory of Separation and Reaction Engineering – Laboratory of Catalysis and Materials, Faculty of Engineering, University of Porto, Rua Dr. Roberto Frias, 4200-465 Porto, Portugal

**Keywords:** saponin extracts, biosynthetic routes, processing, characterization, delivery systems, industrial applications

## Abstract

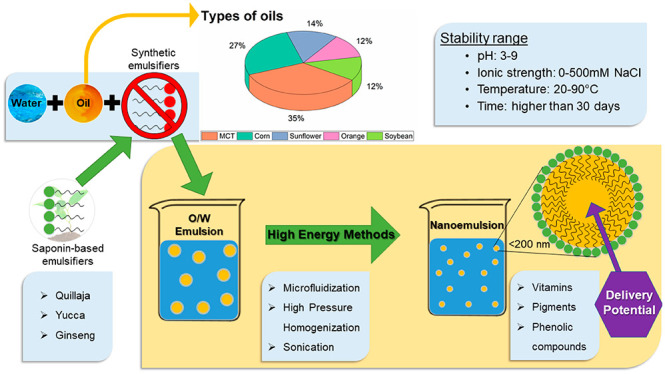

The awareness of sustainability approaches has focused attention on replacing synthetic emulsifiers with natural alternatives when formulating nanoemulsions. In this context, a comprehensive review of the different types of saponins being successfully used to form and stabilize nanoemulsions is presented, highlighting the most common natural sources and biosynthetic routes. Processes for their extraction and purification are also reviewed altogether with the recent advances for their characterization. Concerning the preparation of the nanoemulsions containing saponins, the focus has been initially given to screening methods, lipid phase used, and production procedures, but their characterization and delivery systems explored are also discussed. Most experimental outcomes showed that the saponins present high performance, but the challenges associated with the saponins’ broader application, mainly the standardization for industrial use, are identified. Future perspectives report, among others, the emerging biotechnological processes and the use of byproducts in a circular economy context.

## 1 Introduction

Nanoemulsions are colloidal dispersion systems formed by two immiscible liquids brought into a single macroscopic phase by an emulsifier. These systems possess small-sized particles that can be kinetically stable over long time scales and are least sensitive to physical and chemical changes.^1^ Besides, nanoemulsions are great options to deliver lipid-soluble bioactive compounds designated to increase bioavailability and water-dispersion capability.^2^ Some physicochemical and physiological mechanisms have been proposed to explain the increased bioavailability of encapsulated lipophilic components. One affirms that as the particle size decreases, the solubility of lipophilic components within the aqueous phase surrounding the lipid nanoparticles increase, which can be explained by a thermodynamic effect (Ostwald–Freundlich correlation) associated with the small particle sizes.^3^ In case of nanoemulsions ingestion, a higher concentration gradient of the encapsulated lipophilic constituents from the lipid surface to the cell surface is achieved, resulting in a higher driving force for mass transport and absorption. Also, by decreasing the particle size, the total surface area of the lipid droplets increases, favoring the rate of lipid digestion. Likewise, for topical applications, small droplet sizes can be advantageous since they may pass through the pores (∼400 nm) of the mucous layer, leading to the coating the epithelium cells. Therefore, the lipid droplets may be absorbed directly by the epithelium cells. Thus, lipophilic actives can interact faster and more efficiently with the skin, not clogging the pores, letting the air and water flow between them.^4,5^

The emulsifier plays a crucial role in the formulation of these systems. It reduces the interfacial tension, enhances further droplet disruption, and provides a protective layer around the droplets that improves long-term stability and inhibits their aggregation. The emerging worldwide market of these compounds is projected to reach 8.44 billion dollars in 2021, with an annual growth rate of 6.8% between 2016 and 2021.^6^ This high demand is justified for the vast field of applications and the need to deliver products with higher performance and cost-effectiveness.^7^ Most commercialized emulsion-based products are formulated using synthetic emulsifiers.^8,9^ Because of the enormous scale, a substantial impact on the environment (including toxicity toward living organisms) has been observed, representing risks to the ecosystems and human health. It is of high importance to find the environmental fate of these emulsifiers once they can move freely within the waters, atmosphere, different types of sediments, and even in living organisms.^10^ Currently, there is increasing pressure from consumers for more sustainable and environmentally friendly formulations, in line with increasingly restrictive environmental legislation. Therefore, the search for genuinely biobased emulsifiers that are biocompatible, biodegradable, skin-friendly, chemically inert, highly stable, and consequently, with long shelf lives, are gaining extreme attention.^11,12^ While the nanoemulsion studies were first reported at the beginning of the century, natural alternatives to stabilize these systems have been brought to attention only after 2010.

The replacement of synthetic emulsifiers for natural options is yet challenging. Finding an economically viable source that is generally recognized as safe (GRAS) and presents a consistent functional performance are steps to be most improved. Furthermore, many natural ingredients are highly dependent on environmental factors, where their properties can be considerably changed according to weather, soil conditions, and the time of year they are harvested. Besides, appropriate isolation methods are another topic of concern as the extraction and purification have to be sustainable, scale-up, robust, reproducible, all dependent on the sources and properties of the emulsifier.^9^

Researchers are directly comparing natural with synthetic emulsifiers; Riquelme et al. compared tween 80 and quillaja saponin and concluded that the latter quickly reduced the interfacial tension and increased the electronegativity of the nanoemulsions, indicating that this alternative can form a physically stable system in a vast range of conditions. The interfacial tension reduction using tween 80 was close to 3.6 mN/m, while for quillaja saponin it was around 1.2 mN/m.^13^ The ability of saponins to arrange between different interfaces and for better quantitative analysis, the interfacial and surface tension of systems containing saponins from different sources as well as from some synthetic emulsifiers used at the industrial level are compiled in Table 1. It is clear that the values of the saponins are comparable to synthetic emulsifiers denoting their potential to form and stabilize emulsion systems.

**Table 1:** Surface and Interfacial Tension Using Synthetic Emulsifiers and Saponins from Different Sources

emulsifier	interfacial tension (mN/m)	surface tension (mN/m)	ref
Saponin Sources			
Quillaja	1–7	27–42	(11), (14), (15)
Yucca	3	38	(16)
Ginseng	4–5	37–40	(17)
Tea	5	30–41	(15), (18), (19)
Glycyrrhizin	10–12	59–72	(20), (21)
Red beet	16	29	(22)
Oat bran	7	30	(23)
Synthetic Emulsifiers			
Tween family	2–7	34–45	(15), (24), (25)
Span family	3–4	38–42	(26)
Sodium dodecyl sulfate (SDS)	3	31–65	(9), (27)
Sucrose esters	1–7	35–53	(28)

In fact, saponins are one of the most prominent groups of naturally occurring emulsifiers, whose biological activities (anti-inflammatory, antimicrobial, antioxidant, antiviral, hypocholesterolemic) and physicochemical properties (amphiphilic nature and high surface activity) extend their use to several applications in the pharmaceutical, food, and cosmetic industries. In a general way, saponin-extracts are recognized as secondary metabolites, low-surfactant emulsifiers, being classified as anionic-type, stabilizing the oil droplets by electrostatic repulsion. The saponin molecules contain a triterpenoid or steroid backbone (hydrophobic content) and several saccharide residues (hydrophilic content) attached to the hydrophobic scaffold via glycoside bonds, making them amphiphilic with high surface activity.^29^ Moreover, the abundance of saponins in nature facilitates their commercial production^16,17^ from a wide range of natural matrices, with quillaja bark being the most employed.^11,29^ Furthermore, saponins are also found in waste products, which are promising options due to their cost and sustainability.^15^

The increasing worldwide significance of natural emulsifiers and saponins encourages researchers to conduct detailed reviews on this field. McClements and Gumus provided a comprehensive study addressing several natural emulsifiers for the production and stabilization of emulsions. The saponins were briefly described in function of their physicochemical characteristics (interfacial tension, critical micelle concentration -CMC-, and interfacial rheology) and the range of environmental conditions suitable for their application.^9^ In addition, studies focusing on saponin molecules can also be found, where their structures, extraction, and purification processes were the main treated topics, not approaching the emulsion productions^30^ or their biological activity and performance to stabilize macroemulsions.^31^ Reichert et al. advanced some of these topics but entirely dedicated to saponins from quillaja. Despite these publications providing valuable information on saponins’ advancements, the literature still lacks the production, performance, and application of this natural emulsifier, particularly in the field of nanoemulsions. Most of the reviews dealing with nanoemulsions’ formation, stability, preparation methods, and properties only scarcely address the topic of natural emulsifiers.^1,32−34^ Therefore, this review gives a new perspective on how saponins from different sources can be used to form and stabilize nanoemulsions, the challenges associated with their application, and the suggestion of future perspectives. In this context, several critical topics were chosen for discussion and rationalize the information available in the open literature: the different sources of saponins, the applied oil phase, the production methods, the characterizations involved, the potential delivery of functionalities of the systems, and the current application of saponins in the industry.

## 2 Saponins: Extraction, Purification, and Characterization

Typically, saponins are extracted by using traditional solvent-based methods. Experimental conditions, such as the solvent temperature (∼30–100 °C), pH (acidic conditions are preferable), and composition, directly affect the obtained product. The most common solvents are water, alcohol (methanol or ethanol), and aqueous-alcoholic solutions. Still, the solubility of certain saponins in ether, chloroform, ethyl acetate, *n*-hexane, benzene, and glacial acetic acid has also been reported.^30,35^ The extraction of saponins showed to be a challenge because of its structural variability, namely the one derived from the different substituents in the aglycone moiety. Moreover, the arrangement, number, and orientation of the sugar units, as well as the type of sugar moieties attached to the aglycone, increase complexity. Extra care should also be taken when performing the extractions since saponins can undergo enzymatic hydrolysis during water extractions, while esterification of acidic saponins may also undergo during an alcoholic extraction.^36^ Therefore, the commercial potential of saponins has been pushing toward the development of new process strategies and re-evaluation of the current technologies for their extraction, separation, and purification where microwave-assisted extraction (MAE),^37,38^ ultrasound-assisted extraction (UAE),^39,40^ macroporous resin (MR) separation,^41,42^ foam fractionation,^43^ and continuous chromatography with multizone and multicolumn dynamic tandem techniques^44^ can be highlighted. The MRs are becoming attractive choices to separate and enrich bioactive substances from natural plant extracts, namely saponins, due to their low-cost, high stability, and strong adsorption capacity.^45^

Regarding the saponins’ purity, it can be influenced by the applied extraction parameters, which need to be optimized. For instance, conditions that maximize the extraction yield can decrease the selectivity and, consequently, the purity of the obtained saponins, demanding extra purification steps. The purification is a challenge since the conventional methods of solvent extraction, column chromatography, and preparative thin-layer chromatography, in many cases, cannot effectively isolate saponins.^46^ This occurs because saponins in plants are present with other compounds of similar polarity. Hence, usually, a sequential approach is required. One of the most common purification methods involves partitioning saponins between water and an organic solvent (immiscible with water), for example, n-butanol. Then further purification can be performed using ultrafiltration, adsorption, solvent precipitation, or chromatography.^30^ One new method to obtain saponins of high purity, only scarcely reported, is fermentation. Using high sugar resistant yeast fermentation, the saponin purity was improved, which was accompanied by a significant improvement in CMC and foam stability.^41^ The molecular-trapping in emulsions monolayers is another technique that brought recent attention due to the high purity output that is above 90% with a total recovery of 94%.^46^

The saponins are predominantly glycosides with one, two, or three sugar moieties attached to the aglycone via glycoside bonds, classified as mono, bi, and tridesmosides, respectively. The sugar chains represent the hydrophilic part of the molecule, where the most common monosaccharides found in this context are d-glucuronic acid, l-rhamnose, d-glucose, d-fucose, l-arabinose, d-xylose, d-apiose, and d-galactose. The aglycones (or sapogenins) compose the hydrophobic parts of saponins and may include steroidal or triterpene backbones.^29^ Steroidal skeletons are mainly composed of furostanol or spirostanol forms. Both steroidal and triterpene sapogenins can present several functional groups (−OH, −COOH, −CH_3_), generating a tremendous natural diversity according to the aglycone content. This diversity is amplified by the number and composition of sugar chains. Therefore, the term ‘saponins’ should be recognized as a complex mixture of glycosides with the same or different sapogenin composition, chemical characterization being an important step. Given this context, the most commonly employed technique is liquid chromatography (high-performance liquid chromatography, HPLC, or ultraperformance liquid chromatography, UPLC). However, due to the complexity of saponins, this analysis can be combined with diode array detection (HPLC-DAD),^47^ electrospray ionization tandem mass spectrometry with evaporative light scattering detector (HPLC-ELSD-ESI-MS),^48^ or quadrupole time-of-flight mass spectrometry (UPLC-QToF/MS).^49,50^ According to Savarino et al.,^51^ mass spectrometry methods have, nowadays, achieved the maturity to allow the identification of saponins in plant extracts, even though saponins usually occur as multicomponent mixtures with compounds of similar structure. The nuclear magnetic resonance (NMR) spectroscopy technique (1D and 2D) has also been reported to establish saponin structures.^52,53^ The appropriate chemical characterization of saponins and saponin-rich extracts can also play a key role to determine the composition–properties relationship, helping to evaluate their suitability toward final applications.

Additionally, during processing and storage, the chemical structure of saponins can be modified. For example, the chemical bonds between the sugar chain and the aglycones, and the sugar residues, may undergo hydrolysis, hydrothermolysis, or enzymatic/microbial transformations. These reactions result in the formation of prosapogenins (partially hydrolyzed saponins), aglycones, or sugar residues, depending on the hydrolysis path and conditions. Thus, an appropriate method for material storage is critical for determining their efficient utilization, for example, drying and freezing procedures.^31^

## 3 Sources of Saponins

The abundance of saponins in nature (found in more than 100 families of plants and some marine sources) results in a wide range of natural matrices for commercial production.^29^ They can be found in dicotyledonous plants such as in the seeds of Hippocastani, roots, and flowers of Primulae, roots of ginseng, the bark of quillaja, roots of Saponariae, among others. They are also found in legumes, such as peas, beans, and soybeans, and other plant groups such as yucca and yams. Generally, the cereals are deficient in saponins, but there is an exception with the Avena species (oats), which present a small percentage.^30,31^ The most known sources containing saponins are compiled in Table 2.

**Table 2:** Saponin Content in the Most Known Rich Plant Sources

common name	latin name	saponin content (% wt.)	ref
Alfalfa	*Medicago sativa*	0.14–1.7	(54)
American Ginseng	*Panax quinquefolius*	1.4–5.6	(55)
Chinese Ginseng	*Panax ginseng*	2–3	(31)
Fenugreek	*Trigonella foenum-graecum*	4–6	(56)
Green pea	*Pisum sativum*	0.2–4.2	(57)
Horse chestnut	*Aesculis hipocastanun*	3–6	(30)
Ivy	*Hedera helix*	5.9	(58)
Licorice root	*Glycyrrhiza glabbra*	22.2–32.3	(54)
Mullein	*Verbascum nigrum*	0.06	(59)
Oat	*Avena sativa*	0.1–0.13	(57)
Primula	*Primula spp.*	3.5–15	(60)
Puncturevine	*Tribulus terrestris*	20–40	(61)
Quillaja bark	*Quillaja saponaria*	15–20	(62)
Quinoa	*Chenopodium quinoa*	0.14–2.3	(54)
Butcher’s broom	*Ruscus Aculeatus*	0.11–1.8	(63)
Saffron crocus	*Crocus savitus*	1.2–3.4	(64)
Soapwort	*Saponaria officinalis*	4.4–5.8	(65)
Soybean	*Glycine max*	0.22–0.47	(54)
Sugar beet	*Beta vulgaris*	5.8	(57)
Tea seed	*Camellia oleifera*	13	(57)
Yam	*Dioscorea composite*	4–6	(7)
Yucca	*Yucca schidigera*	10	(66)

Among the different sources, three are highlighted for their performance and a significant number of studies regarding their application in the stabilization of nanoemulsions, namely quillaja bark, yucca, and ginseng.

### 3.1 Quillaja Bark

Quillaja saponins are extracts from the bark of the Quillaja Saponaria Molina tree (its amphiphilic structure is shown in Figure S.1). This type of saponin presents very strong surface activity, exhibiting excellent emulsifying properties.^67^ Quillaja’s stabilization mechanism has been related to the glucuronic acids present in its chemical structure, which shows a highly negative charge at pH 7. As the pH decreases, the negative charge is gradually lost due to the protonation of the carboxyl groups on the quillaja saponin molecules, decreasing their stability.^68,69^

Quillaja saponins have been used to produce highly effective emulsions, with nanosized droplets (*d* < 200 nm) and stability covering a vast range of conditions (pH (2–8), ionic strength (0–500 mM NaCl), and temperature (20–90 °C)).^17^ Besides, their use as emulsifiers in delivery systems has been addressed to fortify foods by incorporating functionalities such as vitamins, flavonoids, fatty acids, among others.^2,70,71^

The capacity to protect oil droplets from aggregation when the lipid phase crystallizes is another quillaja saponin feature, which is essential to prevent partial coalescence in the production of solid lipid nanoparticles or nanostructured lipid carriers.^9^ The CMC of the quillaja saponins, which indicates the minimum amount of emulsifier to the formation of the first micelle, is in the range of 0.1 and 0.8 g/L, at 25 °C,^30,35,72^ while the Tween family (synthetic emulsifiers) has a CMC around 0.014–0.031 g/L, at the same temperature.^73^ Therefore, a well-established pattern is that natural emulsifier needs more quantity to form emulsions, being one of its disadvantages.

**Figure 1:**
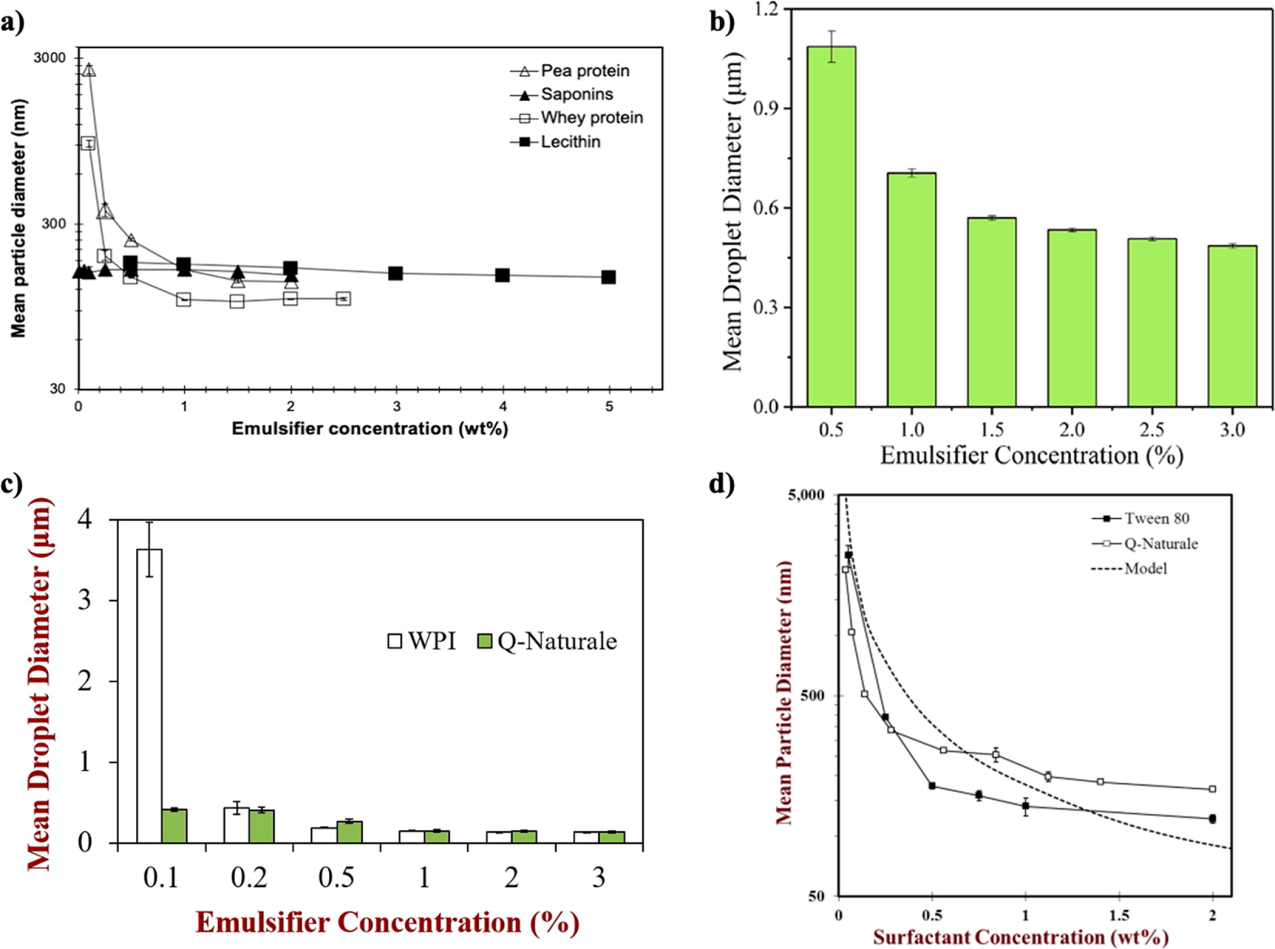
Results for the mean particle diameter varying the percentage of quillaja saponin in 10/90 of oil/water nanoemulsions: (a) Reprinted from Aguiar et al.,^78^ Copyright (2021), with permission from John Wiley and Sons, (b) Reprinted from Lv et al.,^76^ Copyright (2018), with permission from the American Chemical Society, (c) Reprinted from Luo et al.,^75^ Copyright (2017), with permission from Elsevier (WPI corresponding to whey protein isolate), and (d) Reprinted from Yang et al.,^74^ Copyright (2013), with permission from Elsevier.

One of the first works regarding quillaja saponin was performed by Yang et al., which successfully produced oil-in-water nanoemulsions stabilized by Q-Naturale 200 using an air-driven microfluidizer (4 passes under 69 MPa). A comparison with the synthetic emulsifier Tween 80 was also performed. Using interfacial tension studies, the authors concluded that both present equivalent surface activities. Regarding particle size, even if the synthetic emulsifier showed better results, values below 200 nm were achieved applying 1% wt. of saponin. Another result of that work was the influence of emulsifier concentration on the mean particle diameter. Other authors also carried out this study with quillaja saponin, as shown in Figure 1, all using the same oil/water proportion (10/90). As expected, in all cases, it is evident that as the saponin concentration increases, the droplet size decreases. However, the importance of all this data is to show the relevance of finding the minimum saponin amount to achieve the lowest droplet diameter since a constant is achieved between 0.5^75^ and 2% wt.^76^ Additionally, the systematization of these findings by the design of experiments with the representation of response surfaces is an even better option, which our group reported before.^77^

The influence of environmental stresses was also evaluated by Yang et al. for the quillaja saponin, resulting in stable systems in temperature ranges, from 30 to 90 °C, salt concentrations below 300 mM NaCl, and pH conditions between 3 and 8. This stability can be attributed to the ability of the saponin to generate a strong short-range steric repulsion that prevented the droplets from coming close enough to coalesce. However, on low pH and high salt concentrations, destabilization was observed due to the reduction of the electrostatic repulsion, which is the stability mechanism of quillaja, generated between the highly negatively charged droplets. The range of environmental stability was corroborated several times in other works for this saponin.^2,15,71,78,79^

Regarding regulatory aspects, quillaja extracts have been approved in several commissions. Its use as a food additive was allowed under European Commission regulation number 1129/2011/EC. It was also stated as an approved ingredient in cosmetic products at the same commission (regulation number 2006/257/EC). Additionally, the US Food and Drug Administration (FDA) permitted this extract as a food additive and is considered GRAS by the Flavor and Extract Manufacturers’ Association (FEMA). The maximum level of 50 mg/kg (on saponin basis) of quillaja extract was stated by the Codex Alimentarius in its General Standard for Food Additives, to be applied as an emulsifier and foaming agent in water-based drinks.^11,80^

There are a few commercially available saponin products used as an emulsifier, and these options are almost exclusively derived from quillaja.^11^ Q-Naturale (from Ingredion, Bridgewater, NJ) is the leading example for applications within the food industry. The product is provided in two forms, powdered or dissolved within an aqueous solution.^9^ It is important to add that the use of quillaja saponins with other proposes has already been applied. For example, the quillaja saponin has been used as an effective vaccine adjuvant due to its unique profile of immunostimulating activity.^81,82^

### 3.2 Yucca

Yucca saponin extract is obtained from the shrubs and trees of *Yucca schidigera* (Asparagaceae), native to North America. These saponins are steroidal saponins possessing spirostanol and furostanol aglycones. The sugar chains of yucca contain two or three sugars, which can be glucose, galactose, or xylose linked to the aglycone (see Figure S.2).

Valuable insights about the interfacial and emulsifying properties of yucca saponins, as well as their applicability in the food industry, have been demonstrated by the produced nanoemulsions.^16^ The reported CMC of yucca saponin is between 0.01 and 0.1% wt.^16,72^ The emulsions formed with yucca resist a wide range of conditions due to sufficient electrostatic repulsive forces between the droplets. With other environmental stresses applied, such as heating and freeze–thaw treatment, instability has been observed. The instability of emulsions at high ionic strength and low pH was verified, probably due to the reduction of electrostatic repulsion. The ability of this component to form emulsions has been registered in patents,^83−85^ reporting the yucca capacity to be used as an emulsifier agent.

Also, yucca-based oil-in-water (o/w) emulsions formulated with nonpurified sunflower oil were characterized by presenting elasticity 50-times higher than a base emulsion, that is, an emulsion produced with purified sunflower oil. This fact can be considered a powerful new approach to structure o/w emulsions since the elasticity inhibits the syneresis, making the systems homogeneous and with long-term shelf storage stability.^86^

Besides, these extracts present a combination of saponins and polyphenols (e.g., resveratrol, yuccaol, and yuccaone),^16^ which gives strong biological effects such as anti-inflammatory activity, antiplatelet, antioxidant, and antibacterial effects, and growth-inhibitory activity against different yeasts. Besides biological activity, yucca saponins also exhibit significant surface activity. Concerning adsorption layers, the main difference between them and quillaja saponins is that while yucca presents a purely viscous rheological response, quillaja behaves as a two-dimensional viscoelastic body. This can be explained by the molecular structure of their aglycones (steroid vs triterpenoid).^86^

Furthermore, there are two yucca products on the market, the powder (dry form) and the extract. To obtain them, the trunk of the plant (yucca logs) is mechanically macerated, dried, and ground to produce yucca powder, while for yucca extract, the macerated material is subjected to mechanical squeezing in a press, producing yucca juice. Thereafter, the juice is concentrated by evaporation, being the concentrated product referred to as yucca extract. Both possess the GRAS label attributed by the FDA, allowing their dietary use.^87^ It is crucial to highlight that the yucca’ saponins to stabilize emulsions are much less studied than quillaja, proving that stronger efforts are needed in the characterization and search for alternative sources of saponins.

### 3.3 Ginseng

The pharmacological effects of ginseng saponins justify why ginseng is so well-known, namely in Asian herbal remedies and foods, as a valuable ingredient. Their health benefits, such as antiaging, antidiabetes, antifatigue, anticancer, neuroprotective, and hepatoprotective, have been attributed to saponins, which show a diversity of structural compositions.^88^ Besides its functional effects, ginseng also presents high surface activity properties, that is, surface tensions around 40 to 45 mN/m,^89^ because of one or more hydrophilic glycoside moieties and a lipophilic triterpene derivative on their molecular structure. The ginseng saponins, also called ginsenosides, can be divided into neutral or acidic ginsenosides (see Figure S.3). The acidic ones contain dissociable groups such as glucuronic acid and glucose within the sugar moieties, and glucose linked to an ester. The neutrals only have two disaccharides of glucose linked at the same position.^17^

Again, the information on ginseng is not comparable to quillaja, where only a few studies were addressed on its use as an emulsifier. In the production of nanoemulsions, two works addressing ginsengs were identified, reporting *Panax ginseng* L.^16^ and ginseng extracts from leaf and stem,^88^ plus one using the so-called Brazilian ginseng roots, added due to the morphological similarity to ginsengs and richness in saponins (studied parts roots).^90^ All sources demonstrated nanosized systems with high surface activity and good stability, proving their potential performance as emulsifiers.

Ginseng also presents the GRAS label by the FDA.^91^ In the market, ginseng has been introduced as a conditioning agent in the personal care industry^92^ and as an ingredient in energy drinks due to its health benefits.^93^ However, their use as emulsifying agents is still in the early stages.

### 3.4 Other Sources

The sources mentioned above are the most used as emulsifiers in the saponin field, being them directly obtained from nature via extraction. Other examples of this class include puncture vine (*Tribulus terrestris*), fenugreek (*Trigonella foenum-graecum*), and butcher’s broom (*Ruscus aculeatus*).^35^ However, other authors have worked with saponins obtained from agro-industrial wastes or byproducts. Ralla et al. studied the use of food byproducts, red beet (*Beta vulgaris L.*),^22^ and oat bran (*Avena sativa L.*),^23^ concluding that both obtained saponins present the necessary performance to be used as natural emulsifiers. Another example is the argan subproducts, namely the shells and oil press-cake, bioresidues rich in saponins that can act as surface-active components, enhancing droplet stability.^69,94^ Lastly, the *Camellia* seed oil press-cake, which is generally disposed as waste, is a rich source of tea saponin and showed outcomes similar or even better than the extensively studied quillaja saponin, constituting a more sustainable and economically viable source of natural emulsifiers.^15,95^

One should also mention that genetic engineering tools can help, in the near future, to supply the high demand for saponins. To perform the biosynthesis of triterpenoids, three key enzymes are applied, oxidosqualene cyclases, cytochrome P450 monooxygenases, and uridine diphosphate-dependent glycosyltransferases.^96^ Understanding the regulatory mechanisms of the accumulation of triterpenoids in plant, and microbial hosts, and the expression of biosynthetic genes, should permit further promising developments for the production of saponins.^97^ An example is the work of Guo et al.,^98^ where a heterologous production of triterpenoids by introducing various triterpenoid biosynthetic pathways using the *Saccharomyces cerevisiae* was studied. The yield of various triterpenoids was improved from mg L^–1^ to g L^–1^ scales by engineering-related enzymes and yeast metabolism. The authors concluded that metabolic and protein engineering allow further modification of yeast to efficiently produce targeted triterpenoids, leading to their industrial production in opposition to its extraction from plant sources.

## 4 Saponins as Emulsifiers

The high surface activity of the saponin molecules allows their traditional use as natural detergents (e.g., the Camellia oleifera^99^ and Quillaja^31^ based detergents). However, their high-performance potential offers the possibility for its application as emulsifiers in several emulsion fields.

There are many indications that the oil-in-water emulsions produced by saponins show promising results with small droplet sizes and stable to aggregation over a range of conditions.^30,31,74^ Interfacial layers cause their high stability with good dilatational elasticity, inhibiting droplet deformation and coalescence.^29^ Globally, the saponin coated around the emulsions droplets has very good aggregation stability from pH 3 to 8. In the case of large pH values, the high negative charge prevents the droplets from aggregating. However, the droplets become less negatively charged once the pH decreases to a certain level, and flocculation at pH 2 usually happens. When there are high salt levels, the droplets become highly unstable to flocculation due to the reduction of electrostatic repulsion. Systems formed with saponins also have good heat stability. Because of the strong steric and electrostatic repulsion between the droplets, the stability values at neutral pH can go from 30 to 90 °C. Saponin-stabilized nanoemulsions have also proven to be more stable to droplet aggregation in the stomach. They, therefore, have a higher surface area and faster digestion rate in the small intestine, which contributes to its use in food applications.^9^

Some key features helping to describe surface-active compounds include Gibbs energy, contact angle, surface density, size of the polar headgroup, and adsorption time. For quillaja saponin, these properties have been studied, presenting Gibbs energy of adsorption between −36 (at 298 K) and −44 kJ mol^–1^ (at 338 K).^100^ The static contact angle of quillaja saponin was 57°.^101^ Furthermore, the surface density was 0.2 nmol cm^–2^, showing an area per headgroup of 83 Å^2^, higher than the one of a typical nonionic surfactant (e.g., polyoxyethylene alkyl ether, C15E8, 45 Å^2^), being this fact attributed to the number of sugar groups in the saponin hydrophilic portion.^102^ Regarding the adsorption time, Stanimirova et al.^29^ reported 3 s using 0.1% wt. of quillaja saponin, while for Tween 20 the value was 3.1 ms and for SDS 27 μs using the same concentration. This time is also longer than the theoretically predicted value for diffusion-controlled adsorption, meaning that the quillaja saponin adsorption is barrier-controlled around and above the CMC. To the best of our knowledge, no equivalent values are available for other saponin sources. This lack of information points out the need to proceed with studies to improve the rationalization and systematization of these fundamental parameters in the saponin emulsifiers field.

Once there is a wide range of botanical origins, the extracts of saponins possess different compositions. Because of this, it is challenging to identify structure–function relationships. In addition, researchers previously observed saponin extracts from the same botanical origin with different interfacial properties.^102^ Regardless of these aspects, the saponin interest still increases due to its enormous potentialities resulting from the high surface and interfacial activities^14^ and reinforces the importance of more studies concerning the saponin properties, its better characterization through chemical analysis, relating to applications. Several researchers have been advocated to investigate the applicability of saponins from different sources as emulsifiers in the production of nanoemulsions. An overview of such studies is provided in the tabular chart (Table S.1), along with information on lipid phase, preparation methods, molecules to be delivered (or their absence), and a summary of the primary outcomes.

Another tool to interpret and understand their kinetics, self-assembly mechanism, and shape of micelles formed in solutions containing saponins is molecular dynamics (MD).^103^ The structure and conformations in an aqueous solution of quillaja saponin have been reported by Pedebos et al.^104^ The saponin’s aggregation process gradually formed micelles until they reached a plateau in different time frames.^105^ The same saponin was also studied by Magedans et al., suggesting that when the micelles in aqueous solutions are formed, the ester linkage between the fucose residue and the acyl chain is less solvated, it is more protected against degradation, increasing storage stability. Other work^106,107^ performed MD simulations to determine saponins’ conformations in solution and relate the molecular structure with their bioactivity, finding that the central glycosidic bond has a crucial role. The interpretation of rheological characteristics of surfactant adsorption layers at interfaces has already been performed for other systems,^108^ but not for saponins, which is a field to be investigated since it can give information regarding the behavior of these important emulsifiers.^109^

Saponins are being the topic of numerous patents, addressing issues like extraction/purification processes,^110−113^ biological activity (e.g., with immunostimulating and anti-inflammatory potential),^114−116^ and use as adjuvants to enhance absorption of pharmacologically active ingredients.^117−119^ Because of the focus of this review, the patents using saponins as emulsifier agents will be highlighted next. Concerning the topic of saponins use to stabilize emulsions, applications in several industrial fields have been patented. The food sector is the one most explored in several applications. They include, for example, the use of nanoemulsions stabilized by quillaja saponins in the manufacturing of clear beverages, resulting in crystal-clear products maintaining the original flavor.^120^ Also, in the beverage segment, Gillespie^121^ patented the substitution of egg whites in alcoholic and nonalcoholic beverages by quillaja saponin, and Camacho and Lobo^122^ introduced the same saponin in beer to solve problems related with the turbidity caused by proteins without affecting the quality and stability of the beer foam. Another example is the use of a mixture of quillaja saponin and a protein as a new food emulsifier to improve bread dough fermentation and baking.^123^ The combination of saponins with lecithin was also explored in a patent addressing food applications.^124^

In the field of cosmetics, the quillaja saponin was applied in a cosmetic product comprising hyaluronic acid^125^ and in the development of products for the treatment of skin impurities and as hair dye products.^11,126^ Yucca saponin was also referred in two different patents dealing with the formulation of oil-in-water emulsions as base products for cosmetic compositions and pharmaceutical excipients.^83,84^ Both quillaja and yucca saponins were added as emulsifying/foaming agents in an organic toothpaste.^127^ Other saponins, namely glycyrrhizin and soybean, were added to cosmetic formulations, resulting in improved storage stability at different temperatures and also better organoleptic properties.^128^ The ginseng saponins were also used to obtain nanoemulsions for skin-care with antiaging effects, promoting the proliferation of fibroblast and the biosynthesis of collagen.^129^

## 5 Saponins for Nanoemulsion Preparation

It has been scientifically demonstrated and shown in industrial practice that nanoemulsions are very viable, noninvasive, and cost-effective carriers for functionality delivery,^130^ which can be considered a mature technology. Nanoemulsions can present different rheological, optical, and stability features depending on their composition (e.g., oil and emulsifier type), and preparation methods. For example, Gao et al.^95^ by modifying the emulsifier type (tea saponin, whey protein, soy lecithin, tween 80), and concentration (0.5–10% wt.), obtained emulsions with quite different properties in terms of stability and optical appearance. The tea saponin emulsions (above 1% wt.) were, among the studied natural options, highlighted as the more stable nanoemulsions, tolerating environmental stresses especially in long-term storage analysis at 27 and 50 °C, which was not achieved with for the other emulsifiers. Moreover, due to the natural source and diversity of saponins, product engineering requires, in this case, the application of a series of experimental procedures to prepare, validate and test the nanoemulsions. Rationale over the quality of nanoemulsions from different saponin sources is challenging since the number of independent variables such as temperature, pressure, oil to water ratio, the mass percentage of the emulsifier, number of cycles and production method, and the chemical composition of the saponins all play relevant roles. However, the access to some physical-chemistry parameters, GC–MS chemical, and statistical analysis furnish very useful tools for founding heuristics in the design of nanoemulsions.

### 5.1 Screening Studies

Selecting an appropriate emulsifier is a challenging task that can be facilitated if characterization studies are performed. Several techniques can be applied to understand the behavior and performance of an extract or compound, to form and stabilize emulsion systems. The pseudoternary diagram is one example that is used to evaluate the potential of a specific compositional combination of oil/water/emulsifier to form emulsions.^35^ Besides, the diagram can help map the optimal compositions by showing the effect of mass fractions changes of the different constituents on the phase behavior of the system.^131^

Other parameters may include the emulsifying capacity (or activity), and emulsion stability, as proposed by Wang and Kinsella.^35,132^ The foam capacity, stability, and structure are other topics used to characterize surface-active materials. As an example, Bottcher and Drusch^14^ performed the characterization of saponin-based foams for different botanical origins including quillaja, gypsophila, tea, glycyrrhiza, and tribulus. Lastly, one of the most used parameters is the CMC, which is the minimum emulsifier concentration to form thermodynamically stable micelles, and consequently, sharp changes in properties are observed.^133^ The most traditional technique for CMC determination is the surface tension.^134^ However, alternative techniques have been proposed such as conductivity,^14^ spectroscopy,^134^ dye solubilization,^46^ and titration.^133^

**Figure 2:**
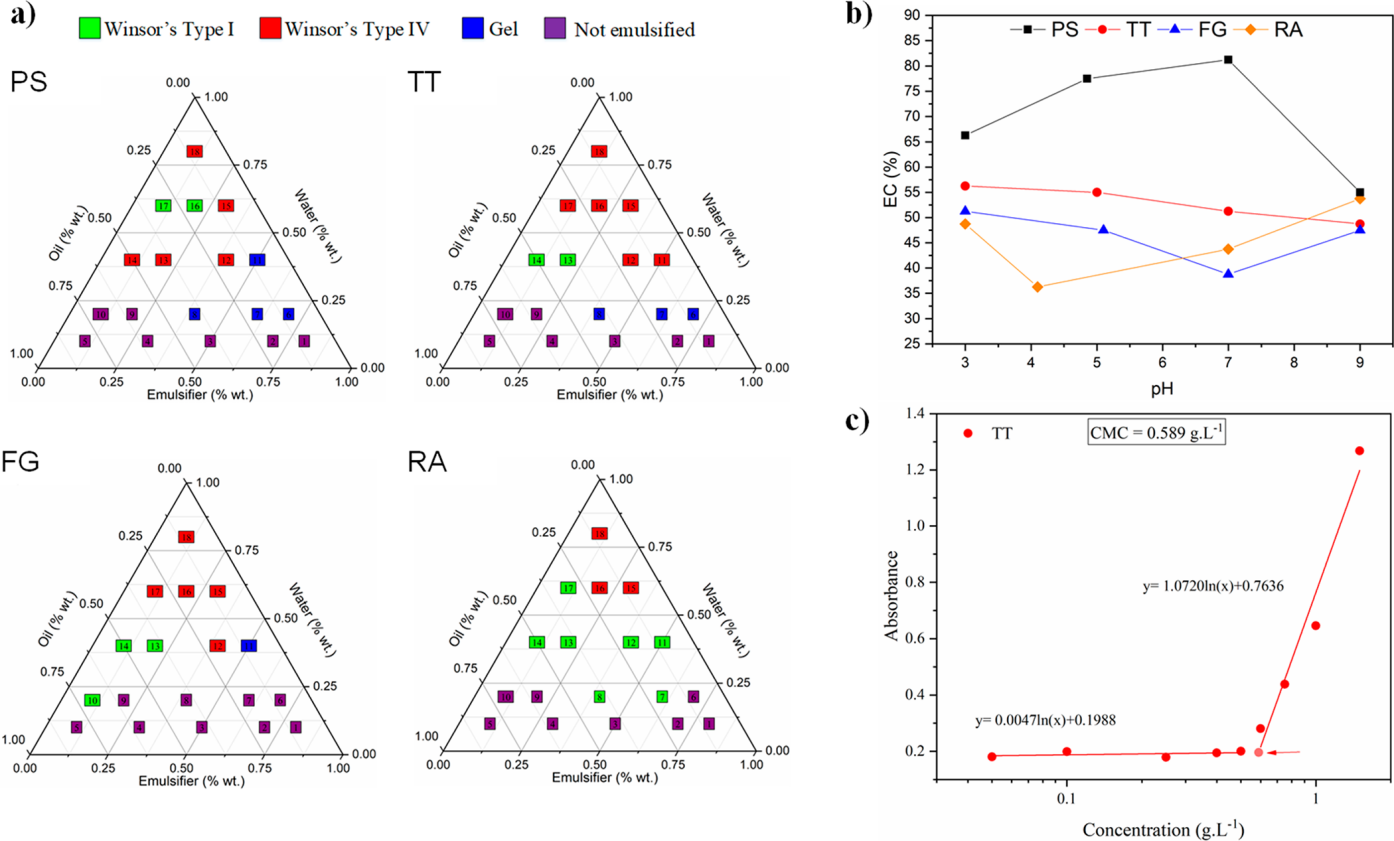
Emulsifiers characterization by (a) ternary phase diagram of pure quillaja saponin (PS), *Tribulus terrestris* (TT), *Trigonella foenun-greacum* (FG), and *Ruscus aculeatus* (RA), (b) emulsifying capacity (EC), and (c) CMC measurement of TT. Reprinted from Schreiner et al.,^35^ Copyright (2021), with permission from Elsevier.

Different saponin-based extracts were subjected to the previously mentioned characterizations by our group,^35^ as one can see in Figure 2. *Tribulus terrestris*, *Trigonella foenum-graecum*, and *Ruscus aculeatus* were positioned as potential emulsifiers, offering advantages over saponin pure forms, holding similar or even additional functional properties.

### 5.2 Lipid Phase

The chemical and physical properties of the lipid phase can be varied by selecting different types of molecules with different structures, molecular weights, or degrees of unsaturation. This selection enables the creation of oil phases with distinct physicochemical properties, such as densities, polarities, refractive indices, and melting behavior. Consequently, it will impact the formation and properties of the nanoemulsions^135^ such as the release and lipid digestion in food applications.^135,136^

Among oil properties, the viscosity can be highlighted for its strong influence on the size reduction process of nanoemulsions droplets. When the relative viscosity of the dispersed and continuous phase is too high, the droplets become resistant to break, which instead start rotation on their axis when subjected to shear. Thus, systems made with high viscous oils have much larger droplets than those fabricated with less viscous ones. If there is a need to use thick oils, the droplet size can be reduced by raising the viscosity of the continuous phase, according to the conditions 0.05 ≤ η_d_/η_c_ ≥ 5, which gives an optimum viscosity ratio between dispersed (η_d_) and continuous phase (η_c_) to produce the finest nanoemulsions.^137,138^

**Figure 3:**
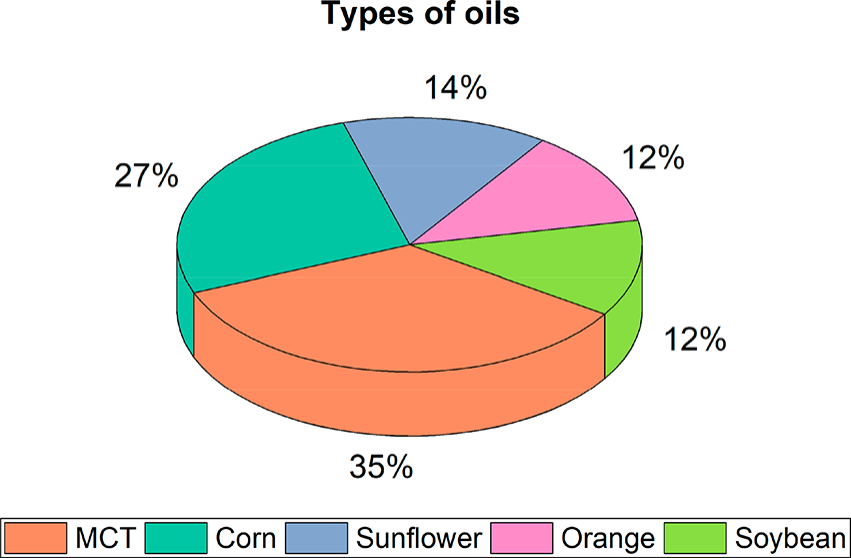
Frequency analysis of the most used oils as lipid phase of nanoemulsions stabilized by saponins.

The relative percentages of the most studied lipid phases in saponin-stabilized emulsions are presented in Figure 3 (sources from Table S.1). The vast majority uses medium-chain triglycerides oil (MCT), that with corn oil sum 62% of the studies. These oils are the most preferred for industrial use. After, at similar proportions, sunflower, orange, and soybean oils, complete the set of the five most used oils for nanoemulsions production with saponins. Several alternative oils have also been studied, such as fish,^68,69,71,139^ lemon,^70^ avocado,^13^ rice,^140^ sweet almond,^77^ among others. Some differences in the magnitude of the electrical charge of the droplets were observed when different oil types were used, which directly impact the systems’ stability.^68^ In a study comparing soybean, sunflower seed, MCT, and orange oils, it was concluded that no significant difference was found when using the first three oils (mean droplet size ∼120–145 nm).^141^ However, the orange oil resulted in much larger droplet sizes (around 800 nm) and showed rapid droplet growth, which was attributed by the authors to the high water-solubility of the oil linked to higher susceptibility to Ostwald ripening. Similar outcomes were reported for other essential oils.^142−144^ One can mention d-limonene, which besides the relatively high water solubility, also tends to oxidize, resulting in the formation of off-flavors. This poor oxidative stability has also been reported for fish oil.^69^

### 5.3 Production Methods

To form nanoemulsions, energy input is needed, where both high-energy and low-energy methods can be applied.^1^ The high-energy techniques are the most used for its production, consisting of preparing a coarse emulsion, followed by intense mechanical force to disrupt the droplets until smaller sizes. Although it is more expensive, the high-energy methods can work with a broader range of ingredients and are become successful in producing nanoemulsions at industrial scale through an easy control of the homogenization devices.^145^ Studies aiming at large-scale production of nanoemulsions were made possible after the spreading of these apparatuses.^146^ The major disadvantage of these methods are related with the energy and instrumental cost, sometimes causing problems for industrial applications. High-energy methods are mainly implemented by one of these three techniques: high-pressure homogenization (HPH), microfluidization, or ultrasonication.^32^

The HPH equipment consists of a pump that pushes the sample through a narrow gap using high pressure between 3.5 and 200 MPa. This procedure causes disruptive forces as shearing, cavitation, and collision. Thus, the coarse emulsion becomes a nanoemulsion due to this hydraulic shear and intense turbulence. The droplet size distribution and mean size are dependent on the pressure, number of recirculation cycles in the system, and temperature, but also the emulsion composition, emulsifier characteristics, and physicochemical characteristics of the different phases influence the final outcome. The advantages of this technique are the easy scale-up, little processing time, with the advantage of avoiding the use of organic solvents. At the same time, some disadvantages include high energy consumption and the difficulty in operating with creamy and high viscous formulations. The HPH is widely used at an industrial level, especially in the cosmetic and pharmaceutic sectors.^33,147,148^

Microfluidization is a technique also using a high-pressure pump (up to approximately 150 MPa). However, it forces the coarse emulsion to pass through some microchannels, called the interaction chambers. Therefore, final nanoemulsion size depends strongly on the number of microchannels and the operating pressure. The droplet size distributions of the nanoemulsions produced by microfluidics are narrower than the ones obtained using other emulsifying devices, due to the nitrogen filtration that removes the larger droplets. Nevertheless, it is also unfavorable in some specific cases, such as when long emulsification times are applied leading to recoalescence and droplet size increase.^33^

Ultrasonication is a very effective method where ultrasound energy (20 kHz and up) disrupts the large droplets into smaller ones. As time increases, energy also increases, leading to the disruption of more droplets, decreasing size. However, there is an optimum limit, where going beyond it is just a waste of energy. The ultrasound technique is a fast and straightforward technique, but it is only appropriate to produce small batches.^137^

**Figure 4:**
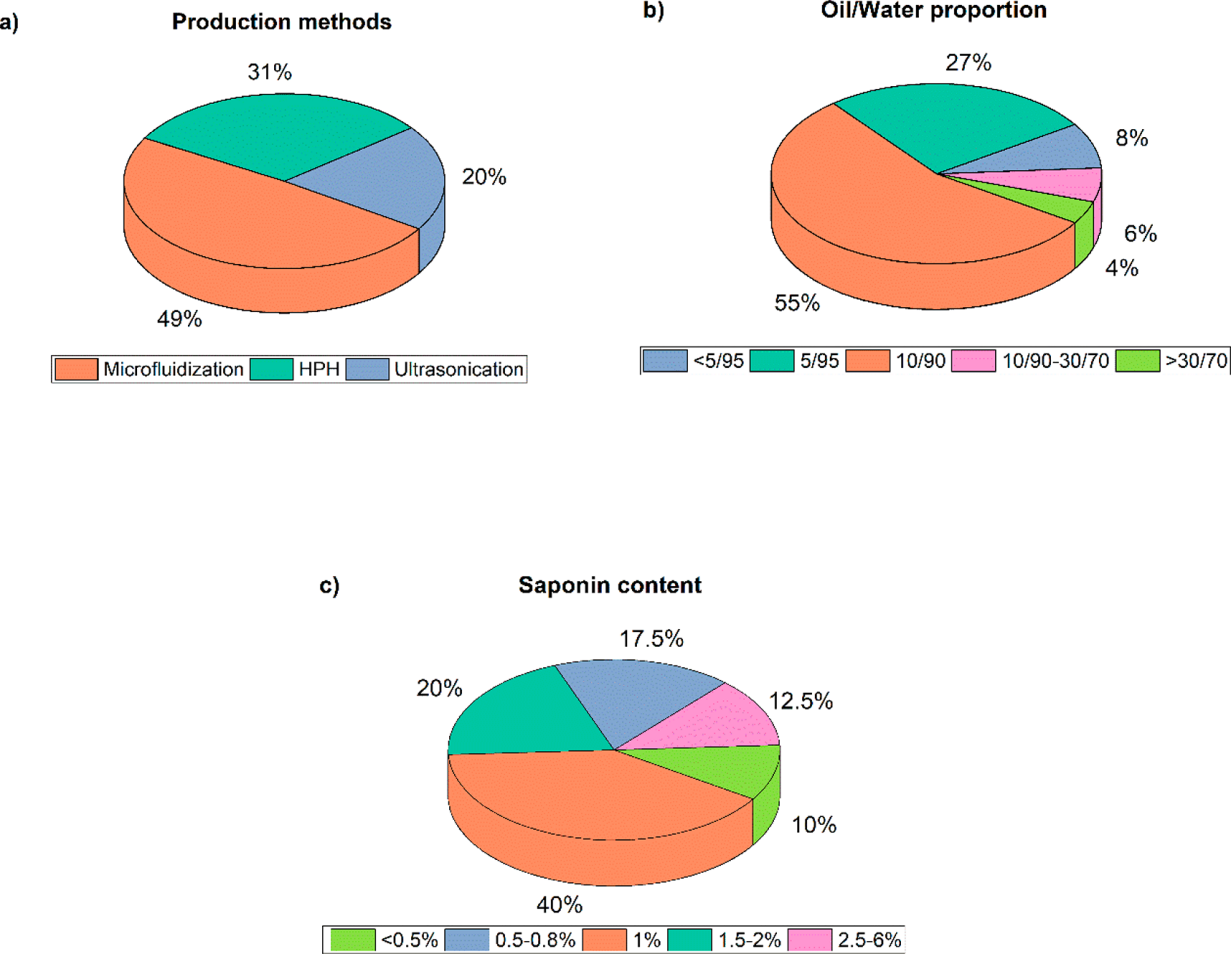
Frequency analysis using saponins in nanoemulsions: (a) production methods, (b) oil/water ratios, and (c) saponin content.

Regarding saponin-based emulsions, the formulations and respective operational conditions for each high-energy methods are summarized in Table 3. The most used device is the microfluidizer, followed by the high-pressure homogenizer. Both methodologies use very similar pressures and number of cycles. The ultrasonication is a less applied technique, probably due to the difficulty in scaling-up. The production of coarse emulsions is a step present in all methods, even sometimes excluded in the microfluidization and sonication, but mandatory in HPH. In addition, the formulations did not show significant differences among fabrication methods, but the vast majority used 10/90% wt. as oil to water ratio and 1% wt. of saponin as emulsifier. Figure 4 presents a comparison between different production methods, oil/water ratios, and saponin contents analyzing the 55 works compiled in Table S.1.

**Table 3:** Formulations and Operational Conditions Applied for Production of Saponin-Based Nanoemulsions Using High Energy Devices

	O/W % wt.	saponin % wt.	coarse emulsion	pressure/*N* of cycles or power/time/pulse	ref
Microfluidization	1/99–50/50 (10/90 is the most common)	0.1–10% (2% is the most common)	High-speed blender for 1–5 min using 6000–24000 rpm (there are works that apply directly microfluidizer^75,76,139,149,150^)	50–150 MPa for 1–8 cycles (4 is the most common)	(2), (67), (143), (144) ,^149−156^, (71), (157−161)(74−76), (78), (79), (139), (142)
High-Pressure Homogenization	5/95–30/70 (10/90 is the most common)	0.1–5% (1% is the most common)	High-speed blender for 1–5 min using 5000–15000 rpm	20–170 MPa for 1–10 cycles (4 is the most common)	(15), (16), (141), (162−166), (17), (20), (23), (68), (69), (88), (94), (140)
Ultrasonication	3/97–30/70 (10/90 is the most common)	0.5–10% (2% is the most common)	High-speed blender for 1–5 min using 6000–24000 rpm (there are works that apply directly sonication^70,167,168^)	Bath and Probe sonication, 10–600 W, 2–50 min, 5–30 s pulse on/off	(13), (70), (90), (95), (167−172)

The low-energy methods are based on the spontaneous formation of emulsions using system conditions to change the interfacial properties. Therefore, it is fundamental to know the emulsifiers’ intrinsic physicochemical properties and use a simple mixing process involving low energy to homogenize the mixture, avoiding breaking the droplets. To the best of our knowledge, only one work attempted the preparation of nanoemulsions stabilized by saponin using a low-energy method, specifically emulsion phase inversion. The authors attributed the unsuccess of the nanoemulsion formation to the poor oil solubility and interfacial characteristics of quillaja saponin.^173^

### 5.4 Nanoemulsions Characterization

Several methods can be used to characterize nanoemulsion systems, with the most common being highlighted in this section.

From a thermodynamic perspective, nanoemulsion systems are unstable dispersions. The destabilization is an unavoidable process, but the time frame can be very large once the process is kinetically more unfavorable than for conventional emulsions.^13^ Therefore, stability studies are performed for assessing this parameter as a function of time and environmental stresses such as thermal processing, pH, ionic strength, moisture, and light. Generally, the nanoemulsions stabilized by saponins presented excellent properties regarding storage time and stability against environmental stresses (Table S.1).

A helpful parameter to predict the stability of nanoemulsions is the zeta potential, which measures the electrical repulsive forces between the droplets, and should be far from zero, that is, higher than 30 mV or lower than −30 mV, to ensure the system stability.^174^ Some authors state that values between −25 and −30 mV still have an energy barrier that can prevent emulsions’ destabilization.^175^ In the case of saponins, due to the carboxylic acid groups in their structure, a negative surface potential is observed. Furthermore, the zeta potential results obtained for saponins indicate that saponin-coated droplets are mainly stabilized by the electrostatic repulsion generated between the highly negatively charged droplets.^69,74,77^ The values obtained for different types of saponins are compiled in Table 4.

**Table 4:** Zeta Potential Measurements of Saponins from Different Sources

saponin sources	zeta potential (mV)	ref
Quillaja	–40 to −60	(16), (77)
Yucca	–25 to −27	(16)
Ginseng	–41 to −57	(17), (90)
Tea	–50 to −54	(15)
Glycyrrhizin	–36 to −50	(20)
Red beet	–39 to −60	(22)
Argan subproduct	–40 to −55	(69)
Oat bran	–50 to −60	(23)

From Table 4, one can highlight the very negative values obtained indicating emulsion’s high stability. The Yucca saponin stabilized emulsions shows the higher values (lower stability), which might be caused by the presence of other surface-active components present in the extract, such as proteins.^16^ These proteins influence the charge properties but still support the formation of stable emulsions, once there is a proven synergistic effect due to the formation of a biogenic saponin–protein complexes that have shown to act as an anchor at the liquid–liquid interface.^14,164^

Other important parameters that help evaluating nanoemulsions’ stability are the droplet size distribution, mean droplet size, and polydispersity index (PDI). Higher stability is achieved with small droplets since they prevent flocculation due to the high curvature and Laplace pressure, avoiding the deformation of large droplets, preventing coalescence trough the formation of a multilamellar film of emulsifier adsorbed at droplet’s interface. Usually, the destabilization of nanoemulsions results from Ostwald ripening. The mean droplet sizes of saponin-based nanoemulsions are also presented in Table S.1.

Viscosity assessment can also be highlighted as a valuable parameter for the physicochemical characterization of nanoemulsions. The results are highly dependent on the composition of the aqueous and oily phases, and emulsifier. Usually, if the water content increases, the viscosity decreases, and as the emulsifier decreases, a more viscous emulsion is produced due to the interfacial tension increases.^32,148^ Because of the saponins’ tendency to form o/w systems, the viscosity of these nanoemulsions is usually low, close to the one of water.

When the nanoemulsions are used as delivery systems, one important aspect to evaluate is the amount of active ingredient entrapped in the formulation. The entrapment efficiency (EE) can be determined from different procedures, including microdialysis technique, gel filtration, dialysis bag diffusion, ultrafiltration, or ultracentrifugation. The technique used to produce the system, the formulation composition, and the nature of the active compound are key factors impacting the EE. For example, Zheng et al.^160^ produced curcumin-loaded nanoemulsions stabilized with quillaja saponin, and depending on the production method, the EE ranged between 55.5 and 93.2%.

### 5.5 Delivery Systems and Alternative Sources

One of the most relevant applications of nanoemulsions is protecting, stabilizing, and delivering functionalities. Their small droplet size associated with their unique physicochemical and functional characteristics reveals their advantages in delivering actives over conventional formulations.^176^

Many active ingredients are lipid-soluble compounds, and their poor water-solubility means that they cannot be directly dispersed within aqueous-based products. Besides, they might be chemically unstable to temperature, oxygen, light, and moisture effects. However, when these components are incorporated at the oil core of oil-in-water nanoemulsions, they can confer protection, preserving bioactivity, improving bioavailability, and providing sustained stability and prolonged release.^145^

So far, considerable research has been conducted on the production of nanoemulsion-based delivery systems, where vitamins,^177−179^ fatty acids,^101,180,181^ and drugs^182−184^ were successfully incorporated. However, most of the developed solutions used synthetic emulsifiers.^185^ Even so, there is now a strong effort in using saponins to produce loaded nanoemulsions.^79,169^

For the first time, Ozturk et al. incorporated vitamin E on nanoemulsions produced with quillaja saponin (Q-Naturale 100), 10/90 of oil/water ratio, with 2% wt. of emulsifier. Different vitamin percentages in the oil phase were used, being 50% wt. identified as the optimum value. Later, Lv et al. also incorporated vitamin E on nanoemulsions formulated with quillaja (10/90 oil/water ratio with 0.5–3.0% wt. of saponin).^76,79^ Besides, the dependence of formation, stability, and vitamin bioaccessibility on the lipid phase composition was analyzed. However, the ideal delivery system was found to be with 20% wt. of vitamin E and 80% wt. of the carrier oil, where the bioaccessibility of the vitamin E remained practically the same after storage (from 54% to 53% after 12 weeks), indicating the effectiveness of this type of delivery system. Comparing both works, there is a big difference in the best vitamin E concentration. One possible explanation for that is the different oily phases selected. Lv et al. used corn oil, one of the most used oily phases, which has already shown great properties in nanoemulsion formation and stability. On the other hand, Ozturk et al. used orange oil, which possesses properties that difficult nanoemulsions stability due to Ostwald ripening (see section 5.3).

The positive outcomes from the Ozturk et al. studies motivated incorporating other functionalities on saponin-based nanoemulsions, mainly in food technology. The incorporation of ω-3 fatty acids^71^ is one example. Nanoemulsions of 1% wt. fish oil (that contains 55% wt. of ω-3), 99% wt. of water and 1.5% wt. of emulsifier were produced by microfluidization. The quillaja saponin was compared to other synthetic (Tween 80 and SDS) and natural (lecithin) emulsifiers, being the one that produce the most oxidative stable systems in the presence and absence of photosensitizers. This may be attributed to the ability of saponin to scavenge free radicals (used in this case riboflavin to promote oxidation) to absorb light in the range that excites them. Thus, the author states that the quillaja saponin could be an outstanding emulsifier for ω-3 ethyl esters nanoemulsions. Other examples of functionalities that were successfully incorporated are ester gum,^151^ quercetin,^70^ β-carotene,^75^ gamma and delta-tocotrienols,^154^ lutein,^159,186^ curcumin,^160^ Origanum essential oil,^144^ carvacrol,^158^ and thyme oil/thymol.^161,169^ As a result, one can conclude that the main groups chosen to be incorporated in the saponin-stabilized nanoemulsions are pigments, phenolic compounds, terpenes, and vitamins. Several of these works focused on food applications, where their characterization is mainly on the bioaccessibility of the active compound in simulations of the gastrointestinal fate.^68,76,79,152,154,155,160,166^ Other characterizations addressed are chemical degradation,^75^ antifungal and antioxidant activity,^161^ colorimetry,^75^ and lipid oxidation.^71^ The common measurements of nanoemulsions’ stability and the effect of the environmental stresses in these systems were also carried out.^2,159^

To the best of our knowledge, to date there are only two studies using two different types of saponins as delivery systems. Khalid and co-workers^166^ used ginseng extract (GS) (80% wt. of saponins)^88^ and gypenosides (GP) (98% wt. of saponins) to produce astaxanthin enriched-nanoemulsions. Astaxanthin is a carotenoid that has beneficial effects on human health and wellness. Both works used 5% wt. of lipid phase (refined soybean oil with 2% wt. of astaxanthin) to 95% wt. of aqueous phase (containing 1% wt. of saponins). The systems were produced by high-pressure homogenization. The volume mean diameter obtained with 100 MPa was 148 nm for GS and 125 nm for GP. Regarding stability tests, the systems were analyzed considering temperature, pH, ionic strength, and time. pH and ionic strength had marked negative impacts on the stability of nanoemulsions, with droplet coalescence occurring for pH values between 3 and 6 and salt molarities above 25 mM NaCl (GS) and CaCl2 (GP). The authors attributed the destabilization to the reduction in the magnitude of the electrostatic repulsion between droplets. It should be highlighted that these conditions are not typical for the destabilization of emulsions based on saponins. For example, another ginseng extract reported stable systems within the pH range of 4–8 and NaCl-addition up to 100 mM.^17^ This outcome can be related to this specific type of ginseng or even the use of astaxanthin. Lastly, in vitro studies were performed for GP nanoemulsions, showing that this saponin provides proper protection against astaxanthin degradation (even better than Tween 20) due to their free radical scavenging ability.

**Figure 5:**
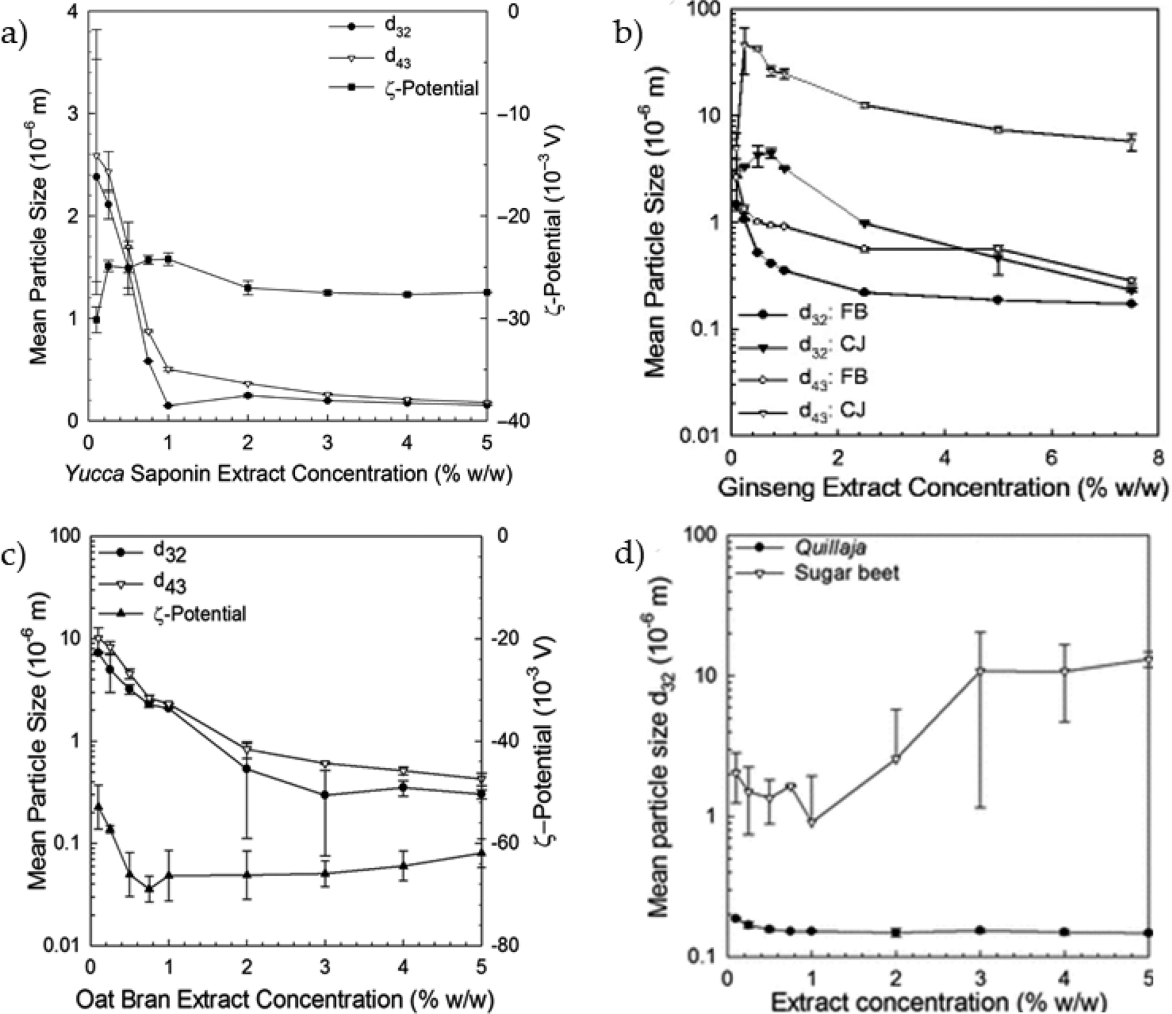
Mean particle size and zeta potential in o/w nanoemulsions (10/90) based on extracts from (a) Yucca. Reprinted from Ralla et al.,^16^ Copyright (2017), with permission from John Wiley and Sons, (b) Finzelberg (FB) and CheilJedang (CJ) Ginsengs. Reprinted from Ralla et al.^17^ Copyright (2017), with permission from Springer Nature, (c) Oat bran. Reprinted from Ralla et al.,^23^ Copyright (2018), with permission from Elsevier, and (d) Sugar beet. Reprinted from Ralla et al.,^165^ Copyright (2017), with permission from the American Chemical Society.

As concluded by the works mentioned above, quillaja saponin represents the most extensively investigated compound, in the area of natural emulsifiers, to produce nanoemulsions and delivery systems. In this context, the search for viable alternative sources of saponins has been gaining attention. Among them, yucca schidigera extract can be mentioned for showing high surface activity and the ability to form nanosized emulsions. Ralla et al. used high-pressure homogenization for the system composition of 10% wt. lipid phase and 90% wt. aqueous phase, and the emulsifier amount varied between 0.1 and 5% wt. Regarding stability studies, it was shown that all nanoemulsions were unstable to monovalent (NaCl) and divalent cations (CaCl_2_), directly contrasting quillaja-based nanoemulsions. However, other environmental studies (temperature and pH) are consistent between the two sources of saponins. Figure 5a shows the influence of the emulsifier concentration on the mean particle size and zeta potential obtained for yucca. The figure also compares other alternative sources, such as (b) two ginseng extracts (Finzelberg: FB; CheilJedang: CJ), (c) oat bran, and (d) sugar beet and Quillaja. In all works, the nanoemulsions contain 10% wt. of MCT oil in their composition. The values of mean droplet size are expressed in *d*_4,3_ and *d*_3,2_, which correspond to volume moment mean, and the surface area mean, respectively, being both important to characterize the power of the emulsifiers. Among the five alternative sources, yucca can be highlighted for reaching the smallest sizes, comparable to Quillaja (Figure 4d), achieving a constant size after 1% wt. of its concentration. For the two ginseng extracts and the oat bran, the curve reaches fairly stable values around 2% wt., while this behavior was not observed for the sugar beet. The authors attributed the difference to the presence of other compounds in the sugar beet extract, where the nonadsorbing surface active components may induce depletion flocculation of the droplets when added at higher concentrations. It is also significant to observe that extracts often require a higher concentration than pure saponin. Possible ways to overcome this difficulty are to control other parameters, such as increased pressure and number of cycles in the preparation or decreasing the formulation’s oil content.

Zhu et al. recently studied another promising option, the tea saponins (TS) isolated from waste materials produced during tea manufacture. This compound may offer a more sustainable and economically viable source of natural emulsifiers. A comparison to the other two emulsifiers (quillaja saponin and Tween 80) was also performed. The chosen proportions were 10% wt. oil phase (MCT oil) and 90% wt. aqueous phase, with an emulsifier amount between 0.1 and 2% wt. The systems were formed by high-pressure homogenization, where TS was slightly more efficient in producing small droplet sizes, achieving the smallest particle size of ∼186 nm, with an emulsifier-to-oil ratio of ∼1:10. Environmental stresses were also investigated, reporting the usual stability range (pH: 3–8, 30–90 °C, and ≤200 mM NaCl). The authors hypothesized that the TS could form a thin layer around the lipid droplets, mainly stabilizing them by electrostatic repulsion. Reducing these repulsions due to environmental conditions (highly acidic, pH 2, or high ionic strength, 300–500 mM NaCl, conditions) causes droplet aggregation.

## 6 Achievements, Challenges, and Future Perspectives

Emulsifiers are the base of several technological products, in areas such diverse as food, cosmetic, pharmaceuticals, and chemical industry. Because of the increasing consumption of emulsifiers several environmental problems are raising concerns related to the accumulation of these silent toxicants in the ecosystem. In such a scenario, a driving force toward their substitution, by green and less ecotoxic solutions are being key areas of research, which include emulsifiers extracted from nature. For the success of the implementation of these solutions, different research fronts have to be pursued, which include diversifying the raw saponin §sources, find alternative ways for their production (e.g., biotechnological routes), invest in more effective, low energy, and economically competitive extraction and purification processes, proceed with their characterization and standardization, and validate their application against the existing synthetic counterparts.

In the context of emulsifiers from nature, saponins draw attention due to their high surface activity and physicochemical and biological properties, postulating them as viable substitutes for synthetic emulsifiers, being extensively tested in the production of emulsions. Globally, the resulting systems showed small particle size (<200 nm), in the range of nanoemulsions, high stability over time (higher than 30 days), including to external environmental stresses (pH, temperature, ionic strength). Commonly, a minimum concentration of the emulsifier (1% wt.) is enough to impart the desired stability. The used lipidic phase includes almost 20 different oils, with MCT and corn oil being preferred due to the lower viscosity, easy access, and low cost. Most used methods, include microfluidization and high-pressure homogenization, with this last presenting higher industrial implementation, even with the disadvantage of being a high-energy device. In this way, alternatives such microfluidization, which are successively approaching full industrial use, have the advantages of higher reproducibility, also giving rise to narrow size droplet distributions.

Despite saponin excellent performance, still some constraints exist for their full industrial application, starting with their extraction. The purity of saponin extracts is highly affected by the presence of the natural matrices’ impurities, namely phenols, fats, tannins, proteins, and sugars, which can demand investment in subsequent purification steps, if high pure saponins are requested, with a consequent inherent cost increase of the product. The saponin content of commercially available extracts could vary between 20 and >97%, demanding investment in testing lower content saponin extracts, which might benefit from the synergist presence of other emulsifying compounds in the mixture. The characterization of these surface-active compounds (interfacial tension, emulsifying capacity, and CMC) is an important step for comparison purposes and, together with chemical characterization, can help standardization, a vital issue for industrial application. In fact, saponin chemical profiles depend on the plant source, plant part, plant stage of development, and collecting region.

Most saponin studies are focused on extracts isolated from the quillaja Saponaria tree due to its great efficacity as an emulsifier, with the advantage of being already commercially available. However, the overexploitation of quillaja has resulted in a tremendous reduction of their natural tree population, a reason why it urges to diversify to other saponin sources. Moreover, economic viability for the use of these natural emulsifiers demands their obtention at relevant quantities, but at a reasonable cost, compatible with their industrial use, for which the design of more sustainable and economically competitive processes should be done. Among them, it is highlighted the emerging use of biotechnological processes to produce saponins and the use of by products in a perspective of circular economy. Also, it is expected that the use of biotechnological processes will also help to circumvent the problems associated with saponins standardization for industrial uses.

Regarding their practical application, the sensory aspect of saponins can be a drawback. Among them, the yellowish or brownish pigmentation and the astringent or bitter taste of saponins were pointed out. In this way, final studies dealing with product development in closed symbiosis with industry and consumers must be carried out to validate their application, which is expected to vary depending on the chosen area. In fact, and face to the published evidence and industrial use, the cosmetic sector seems to be the area of election for the use of these saponin-based emulsifiers. This area can also benefit from the emerging studies dealing with saponin-based emulsions functionalization, which can act as promising delivery systems, for example, for lipophilic vitamins, improving skin absorption of these compounds and thus their efficacy.

Overall, even the significant challenges in the field of saponin emulsifiers such as obtainment and applications, their industrial use, namely in the production of emulsions, products of high technological importance for several industrial fields, it is expected a market evolution in the availability and commercialization of these type of products, preferably following sustainable approaches avoiding deforestation.

## Supporting Information Available

Molecular structures of different saponins; compilation of previous studies on nanoemulsions production using saponins (PDF)
