# Supplementary material for: Saponins as Natural Emulsifiers for Nanoemulsions
Source: J Agric Food Chem. 2022 May 27;70(22):6573–90. doi: 10.1021/acs.jafc.1c07893 (PMC9776534; doi:10.1021/acs.jafc.1c07893)
Supplement: Supplementary file 1 — jf1c07893_si_001.pdf [file jf1c07893_si_001.pdf]

Supporting Information for

Saponins as Natural Emulsifiers for Nanoemulsions

Tatiana B. Schreiner[a,b], Madalena M. Dias[b], Maria Filomena Barreiro[a,*], Simão P. Pinho[a,*]

[a] *Centro de Investigação de Montanha (CIMO), Instituto Politécnico de Bragança, Campus de Santa Apolónia, 5300-253 Bragança, Portugal.*

[b] *LSRE-LCM - Laboratory of Separation and Reaction Engineering – Laboratory of Catalysis and Materials, Faculty of Engineering, University of Porto, Rua Dr. Roberto Frias, 4200-465 Porto, Portugal.*

Structures of Different Saponin Sources

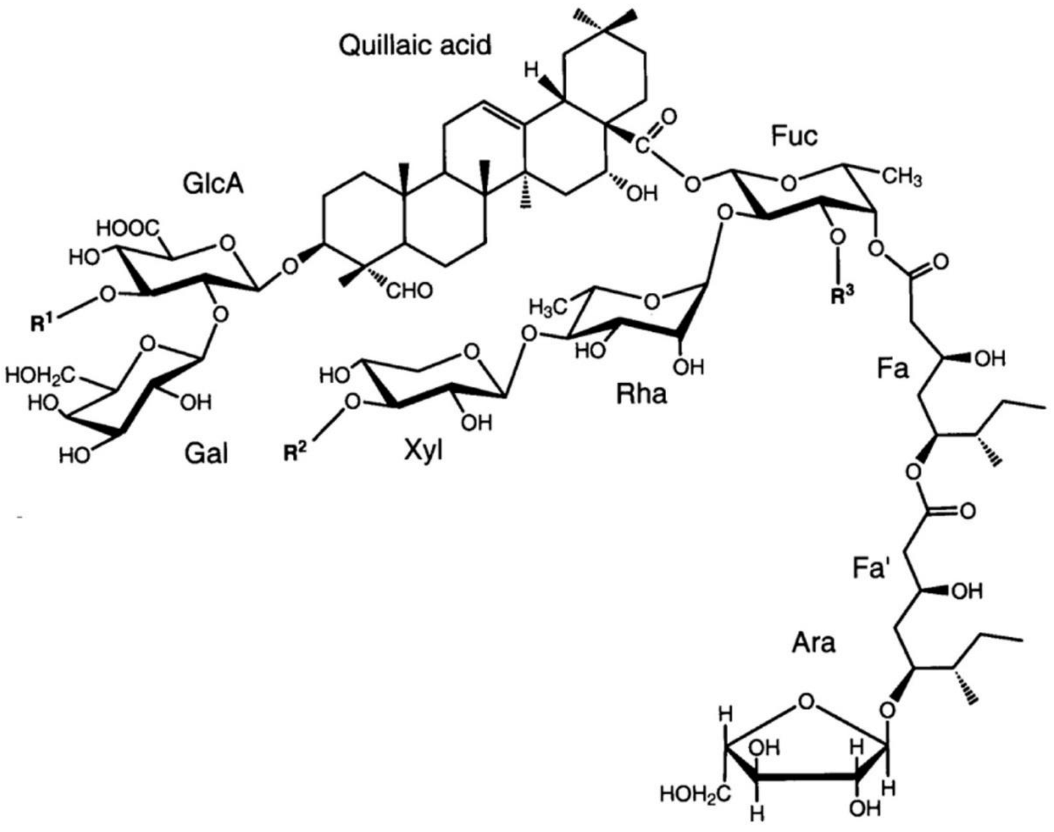

Figure S.1. Molecular structure of bidesmosidic saponins extracted from Quillaja saponaria Molina. Reprinted from Böttcher et al, 2017[1], Copyright (2017), with permission from Elsevier.

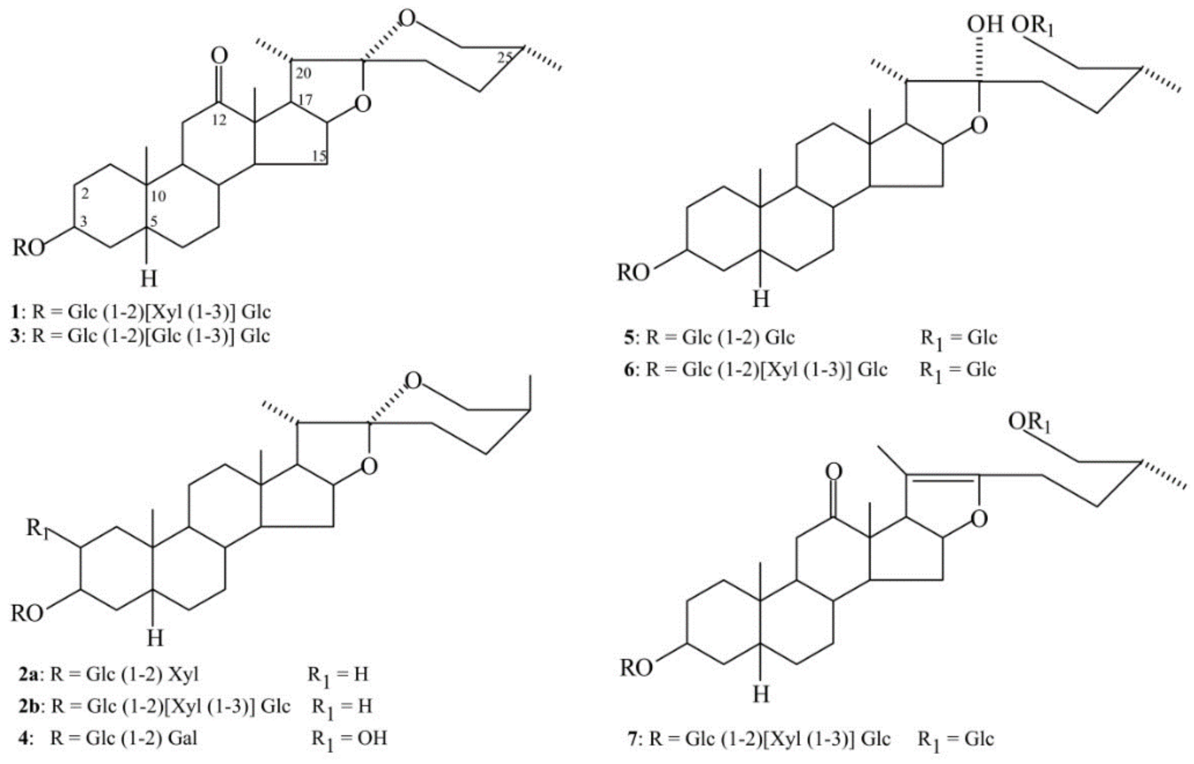

Figure S.2. Chemical structures of yucca saponins from Yucca schidigera bark. Compounds 1–4 represent monodesmosidic and 6–7 bidesmosidic structures. Reprinted from Cheeke et al, 2017[2], Copyright (2006), with permission from BioMed Central Ltd.

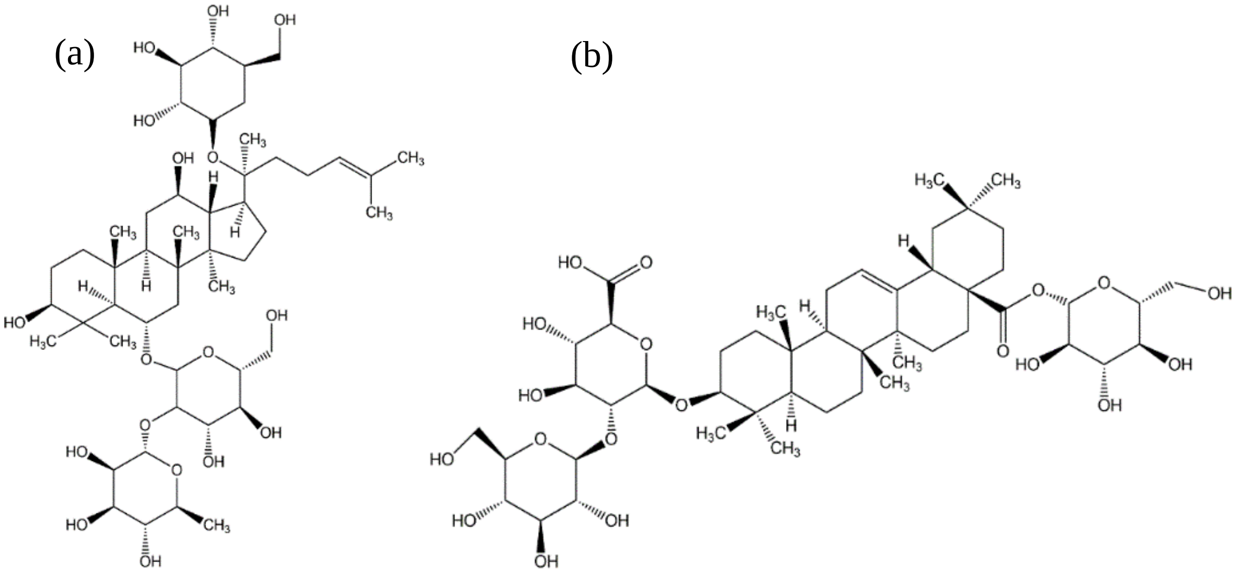

Figure S.3. Molecular structure of ginseng saponins with (a) neutral and (b) acid behavior. The structures were generated in ChemDraw adapted from Shu et al., 2018[3].

Table S.1. Previous studies of the nanoemulsions produced by different types of saponins as emulsifier.

**Saponin Type as emulsifier**	**Lipid Phase**	**Preparation Method**	**Delivery**	**Outcomes**	**Ref.**
Quillaja saponin (Q-Naturale®)	Corn oil	Microfluidization	Capsicum oleoresin	Nanoemulsions (d < 200 nm) were successfully fabricated, being stable to capsaicin degradation when stored for 15 days. Saponin's systems were stable over a range of temperatures (40–90 °C), salt concentrations (≤ 200 mM NaCl), and pH values (pH 3–8).	4
Purified glycyrrhizin and non-purified glycyrrhizin-rich extracts from liquorice root (Glycyrrhiza glabra)	Soybean oil	High-Pressure Homogenization	-	Liquorice root extract with high-surface activity showed to be suitable to produce O/W nanoemulsions. Due to this performance, there is no need to perform extensive purification.	5
Crude saponins isolated from onion skin waste	Sunflower, Soybean, MCT, or orange oil	High-Pressure Homogenization	-	This new natural emulsifier based on onion waste showed high surface activity, minimizing the interfacial tension and producing nanodroplets below 150 nm. However, it demonstrated poor stability at acidic pHs and high ionic strength, which can be attributed to the decrease of the electrostatic repulsion.	6
Quillaja saponin (Q-Naturale®)	Olive oil	Ultrasonication	Cannabis extract	The translucent loaded nanoemulsions with droplet sizes below 100 nm were only possible with synthetic emulsifiers (Tween/Span mixture) but not with the saponin.	7
Saponaria officinalis and Quillaja saponaria	Hempseed oil	Ultrasonication	-	The extracts of Saponaria were found to be better than quillaja extracts in terms of their emulsion stability, centrifugation stability, particle size, and surface tension	8
Pure Saponin from *Quillaja* Bark	Sweet almond oil	High-Pressure Homogenization	-	Nanoemulsions were successfully produced with long-term stability. From the design of experiments, most attract formulations indicate a 10/90 O/W ratio and 1.5% emulsifier.	9
Yucca Schidigera saponin	Sunflower oil	Colloid mill	-	O/W emulsions were successfully formed and remained homogeneous and stable after 30 days of storage. They presented a highly elastic mixed-adsorption layer on the surface of the oil drops, with elasticity 50 times higher than conventional emulsions.	10
Argan shells extract	Soybean oil	High-Pressure Homogenization	-	The extract was capable of producing submicron emulsions with a highly negative charge and good physical stability. The composition of the extracts contributes to emulsion formation when using complex mixtures of surface-active compounds, and emulsion trials have to be conducted to reveal this tendency.	11
Saponin (Merck)	Thyme essential oil	Mixing and autoclave	-	Nanoemulsions were effectively produced with small particle sizes and PDI. Also, it presented high antioxidant activity and high antibacterial and antifungal activities against *Staphylococcus aureus* and *Penicillium digitatum*.	12
Tea saponin	Virgin coconut oil and green tea seed oil	Ultrasonication	-	The tea saponin could form and stabilize nanoemulsions with a smaller droplet size than other emulsifiers, such as whey protein and soy lecithin, and close in size to the synthetic Tween 80. Besides, they maintain good stability over pH, temperature, and time changes.	13
Quillaja saponin (Q-Naturale®)	Clove oil and corn oil	Microfluidization	-	Physically stable clove oil nanoemulsions could be fabricated with saponin by using high-pressure homogenization. Nanoemulsions could inhibit fungal growth and DON production during the micro-malting process.	14

Quillaja saponin (Q-Naturale®) with Rice glutelin	Refined rice bran oil	High-Pressure Homogenization	-	Formed emulsions with relatively small droplets by mixing both components. They showed good stability to high salt levels and temperatures. Both steric and electrostatic repulsion are essential contributors to the stability of the mixed emulsions.	15
Quillaja saponin	Thymol oil	Ultrasonication	Zinc ($ZnSO_4$)	The nanoemulsions loaded with zinc were successfully produced and could be an alternative to synthetic agrochemicals and used for sustainable agriculture coupled with protection of the biosphere.	16
Tea saponin	MCT oil	High-Pressure Homogenization	-	Nanoemulsions (d < 200 nm) could be fabricated from tea saponins at relatively low emulsifier-to-oil ratios. Oil droplets coated with these emulsifiers were stable over a range of temperatures (30–90 °C), salt concentrations (≤ 200 mM NaCl), and pH values (pH 3–8).	17
Saponin (pharmaceutical grade)	Refined sunflower oil	Ultrasonication	-	The efficacy of saponin was confirmed as a natural emulsifier, with stable nanoemulsions and nanogels successfully produced by using long-chain triglycerides.	18
Quillaja saponin (Q-Naturale® 200)	Corn oil	Microfluidization	Thyme oil	Thyme oil nanoemulsions were positively produced by using a novel electro-hydraulic microfluidizer. The droplets diameter was below 200 nm and presented stability for 28 days of storage. The systems showed high inhibition of mycelial growth and spore germination by disrupting hyphae and spore morphology.	19
Quillaja saponin	Avocado oil	Ultrasonication	-	The replacement of Tween 80 by quillaja saponin as an emulsifier can lead to an effective emulsifying mixture that can form a physically stable nanoemulsion in a wide range of processing conditions.	20
Quillaja saponin (Ingredion)	High oleic sunflower oil and MCT oil	Ultrasonication	Thymol	Thymol emulsions with long-term stability containing a low content of green solvents were successfully produced. Antioxidant assays (DPPH, FRAP, and CUPRAC) showed that the emulsification improved the antioxidant activity of thymol if compared to free thymol.	21
Quillaja saponin (Q-Naturale® 200)	Corn oil	Microfluidization	Vitamin E	Oil-in-water emulsions were successfully produced, with good storage stability at room temperature and resistance to droplet aggregation from pH 2 to 8.	22
Quillaja saponin (Q-Naturale® 200)	Corn oil	Microfluidization	Curcumin	Nanoemulsions developed gave bioaccessibility that was similar to those of the curcumin commercial formulation tested. However, these systems were formulated entirely from natural ingredients, which may be beneficial for consumer applications.	23
Quillaja saponin (Q-Naturale® 200)	Corn oil	Dual-Channel Microfluidization	Vitamin E	Plant-based vitamin E fortified nanoemulsions were successfully produced. The bioaccessibility of the vitamin was not affected when stored for 12 weeks in refrigerated conditions, which suggests that this type of delivery system may be effective in delivering vitamin E.	24
Quillaja saponin (Q-Naturale® 200)	Corn oil	Air-driven microfluidization	Lutein	Emulsions stabilized by quillaja saponins had good physical and chemical stability compared to formulations with other natural emulsifiers, such as whey protein or caseinate. Lutein could be encapsulated in emulsion-based delivery systems formulated from only natural ingredients, such as natural emulsifiers and natural antioxidants.	25
Gypenosides (saponins 98%)	Refined soybean oil	High-Pressure Homogenization	Astaxanthin	Nanoemulsions were effectively produced with small droplet size and excellent stability. The encapsulation of Astaxanthin was successful within nanoemulsion delivery systems.	26

Quillaja saponin	Thymol	Ultrasonication	-	Stable thymol nanoemulsion by sonication were produced with saponin as emulsifier. Strong antibacterial activity, significant disease control, and plant growth promotory activity were achieved with stability of 3 months.	27
Quillaja saponin (Ingredion)	High oleic sunflower oil	Microfluidization	*Origanum compactum* essential oil	The formulation of essential oil nanoemulsions was effective by using saponin. The systems were stable against stress conditions, such as salt and acidic pH. Results show applicable potential for different products, including foods and pharmaceutical products.	28
Quillaja saponin (Q-Naturale® 200)	MCT oil	Microfluidization	Carvacrol	Nanoemulsions formulated with saponin had higher antimicrobial activity than those formulated with Tween 80, indicating that the formulation of delivery systems can influence the measured antimicrobial effectiveness.	29
Oat bran extract	MCT oil	High-Pressure Homogenization	-	Oat bran extract formed submicron-sized emulsion droplets at a low emulsifier-to-oil ratio due to its high surface-active property. Emulsions produced were resistant at a range of pH, heat, and time-induced stresses.	30
Ginseng extract (saponins 80%)	Refined soybean oil	High-Pressure Homogenization	Astaxanthin	Ginseng could successfully formulate Astaxanthin-loaded nanoemulsions. The stability was not affected by thermal treatment, while acidic conditions and high salt levels impact physical stability.	3
Quillaja saponin extract (Andean QDP Ultra Organic)	MCT oil	Microfluidization	-	Oil-in-water emulsions were successfully created. The combination of saponins with other natural co-emulsifiers showed that specific binary emulsifier mixtures could tune the stabilization and interfacial properties.	31
Quillaja saponin (Q-Naturale® 200)	MCT oil	Microfluidization	-	The important role of oil droplet concentration on the appearance, viscosity, and stability of model liquid creamers stabilized with the plant-based emulsifier (saponin) has been shown.	32
Extract of Argan oil press-cakes	Soybean oil, MCT oil, fish oil or D-limonene	High-Pressure Homogenization	-	Successfully created nanoemulsions with narrow droplet size and good physical stability. However, it showed instability with salt addition and extreme acidic pH.	33
Quillaja saponin (Q-Naturale® 200)	Corn oil	Microfluidization	Vitamin D	Nanoemulsification significantly improved the absorption of vitamin D from both in vitro and in vivo studies. There was an improvement in bioavailability and homogeneity of the vitamin that, besides absorption, also reduce the risk for people being exposed to access vitamin D.	34
Quillaja saponin (Q-Naturale® 200)	MCT oil	Microfluidization	Gamma and delta tocotrienols	Tocotrienols were effectively encapsulated within nanoemulsion-based delivery systems. One important consideration is by increasing the overall droplet concentration, a change in the color and shear viscosity is seeing.	35
Korean Ginseng extracts	MCT oil	High-Pressure Homogenization	-	Extracts were highly surface-active and reduced the interfacial and surface tension to values similar to those found in quillaja. They formed nano-sized emulsion droplets.	36
Yucca saponin extract	MCT oil	High-Pressure Homogenization	-	Yucca saponin extract was highly surface-active and could form nano-sized emulsions. Results showed stability over a wide range of pH but showed some instability upon heating.	37
Quillaja saponin (Q-Naturale® 200)	Corn oil	Dual-channel microfluidization	β-carotene	β-carotene enriched nanoemulsions (diameter <200nm) stabilized by a natural emulsifier can be fabricated using this approach. Good stability was seen, with a small amount of aggregation when stored at 55ºC.	38

Sugar beet extract	MCT oil	High-Pressure Homogenization	-	The extract showed high surface activity, and the formation of small emulsion droplets was successful. The work shows that it is an effective natural emulsifier that may be utilized for various food and beverage applications.	39
Quillaja saponin (Q-Naturale® 200)	MCT oil	Microfluidization	-	Saponin was capable of form stable oil-in-water emulsions with small droplet sizes. Besides, it showed to be suitable for applications in liquid coffee whiteners that are stable as is and in hot acidic, high mineral content environments.	40
Quillaja saponin (Q-Naturale®)	MCT oil	Dual-channel microfluidization	-	Concentrated oil-in-water emulsions stabilized by single and mixed natural emulsifiers were successfully fabricated. The droplets' relatively small size and narrow polydispersity were formed from the mixed emulsifiers, which led to higher viscosity.	41
Quillaja bark saponin (mixture with β-lactoglobulin)	Sunflower oil	High-Pressure Homogenization	-	Relatively small droplets (average diameter < 500 nm) were formed, which indicates that solutions were effective in producing nanoemulsions under the evaluated conditions. Good stability of resulted emulsions can be attributed to the increased electrostatic repulsion and steric stabilization conferred by the two emulsifiers concurrently.	42
Quillaja saponin (Q-Naturale® 200)	MCT oil	Microfluidization	-	The potential use of quillaja saponin as a natural emulsifier to stabilize liquid creamers suitable for application in hot coffee drinks was showed. The high stability of the system was attributed to a relatively high negative zeta potential.	43
Quillaja saponin extract	Corn oil	High-Pressure Homogenization	-	The work showed the impact of the composition of quillaja saponin extract on antioxidant activity. The native quillaja saponin extract showed higher antioxidant activity than the purified extract in aqueous media.	44
Quillaja saponin (Q-Naturale® 200)	Fish oil	Microfluidization	-	Physically stable emulsions with high fish oil concentrations (30 - 40 % wt.) could be produced using saponin as emulsifier.	45
Pure saponin (Sigma-Aldrich)	Lemon oil and corn oil	Ultrasonication	Quercetin	Saponin and Tween 80 stabilized nanoemulsions were prepared by the sonication method. The incorporation of Quercetin showed a significant effect on degradation, solubilization, loading, and release properties.	46
Saponin (Sigma-Aldrich Co.)	Vitamin A and E	High-Pressure Homogenization	-	Saponin-based emulsification of vitamins A and E has been shown to be a stable and efficient delivery system for fat-soluble vitamins. Also, it improves the protective effect against oxidative stress-induced cellular damage.	47
Quillaja saponin (Q-Naturale®)	Corn oil	Dual-channel microfluidization	-	Q-Naturale was effective in form nanoemulsions. Small uniform droplets and good stability were achieved by passing just once through the microfluidizer.	48
Saponins from Quillaja bark (Sigma-Aldrich)	Sunflower oil	Microfluidization	Vitamin E	Enriched nanoemulsions were successfully produced with small droplet sizes (< 300 nm). After long-term storage, under different thermal conditions, the nanoemulsions were physically stable with a slight increase in the mean particle diameter.	49
Brazilian ginseng roots (BGR)	Annatto seeds oil	Ultrasonication	-	Prepared emulsions presented a small droplet size, which enhanced the stability of these emulsions. Results showed that BGR saponin might be an attractive biosurfactant choice for emulsion formulations.	50
Quillaja saponin (Q-Naturale® 200) with ester gum	Orange oil	Microfluidization		Quillaja saponins demonstrated exemplary performance in producing flavor nanoemulsions. A small droplet size (69 nm) was achieved.	51

Quillaja saponin (Q-Naturale® 200)	Fish oil ethyl ester	Microfluidization	Omega-3 fatty acids	Omega-3 fortified nanoemulsion could be produced and stabilized using the natural emulsifier. The results showed good stability with pH variation (3-8), salt concentration (≤ 500 mM NaCl), and thermal processing (90°C).	52
Quillaja saponin (Q-Naturale® 200)	Orange oil and MCT oil	Microfluidization	-	Orange essential oil-based emulsion was successfully formulated, allowing the manufacture of good nanoemulsions in beverage and mouthwash applications.	53
Quillaja saponin (Q-Naturale® 100)	MCT, corn oil, fish oil, mineral oil, and orange oil	High-Pressure Homogenization	vitamin D_3	Corn and Fish oils used as lipid phase were found to be the most suitable for increasing the bioaccessibility of vitamin D_3.	54
Quillaja saponin (Q-Naturale® 100)	Orange oil	Microfluidization	Vitamin E	Formation and stability of vitamin E-enriched nanoemulsions were successfully produced. They were stable over a broader range of pH values (3–8) and salt concentrations (≤ 300 mM NaCl) but exhibited flocculation at lower pH and higher salt conditions.	55
Quillaja saponin (Q-Naturale® 200)	MCT oil	Air-driven microfluidization	-	Nanoemulsions were effectively produced with small droplet sizes (d<200nm) at low emulsifier-to-oil ratios, which were stable to a range of thermal treatments (30-90°C), salt concentrations (≤300 mM NaCl), and pH conditions (pH 3-8).	56
Quillaja saponin (Q-Naturale®)	MCT and orange oil	Emulsion phase inversion (EPI)	-	It was not possible to form nanoemulsions using the EPI method (low energy) from label-friendly emulsifiers, such as quillaja saponin, which was attributed to their low oil solubility and interfacial properties.	57

References

(1) Böttcher, S.; Drusch, S. Saponins — Self-Assembly and Behavior at Aqueous Interfaces. *Adv. Colloid Interface Sci.* **2017**, *243*, 105–113. https://doi.org/10.1016/j.cis.2017.02.008.

(2) Cheeke, P. R.; Piacente, S.; Oleszek, W. Anti-Inflammatory and Anti-Arthritic Effects of Yucca Schidigera : A Review. *J. Inflamm.* **2006**, *3* (6), 1–7. https://doi.org/10.1186/1476-9255-3-6.

(3) Shu, G.; Khalid, N.; Chen, Z.; Neves, M. A.; Barrow, C. J.; Nakajima, M. Formulation and Characterization of Astaxanthin-Enriched Nanoemulsions Stabilized Using Ginseng Saponins as Natural Emulsifiers. *Food Chem.* **2018**. https://doi.org/10.1016/j.foodchem.2018.02.062.

(4) Aguiar, A. C. De; Martínez, J.; Paula, J. T. De; Luis, J.; Mundo, M.; Mcclements, D. J. Influence of Type of Natural Emulsifier and Microfluidization Conditions on Capsicum Oleoresin Nanoemulsions Properties and Stability. *J. Food Process Eng.* **2021**, *44* (4), 1–13. https://doi.org/10.1111/jfpe.13660.

(5) Taarji, N.; Bouhoute, M.; Fainassi, F.; Hafidi, A.; Kobayashi, I.; Neves, M. A.; Tominaga, K.; Isoda, H.; Nakajima, M. Interfacial and Emulsifying Properties of Purified Glycyrrhizin and Non-Purified Glycyrrhizin-Rich Extracts from Liquorice Root (Glycyrrhiza Glabra). *Food Chem.* **2021**, *337* (March 2020), 127949. https://doi.org/10.1016/j.foodchem.2020.127949.

(6) Dahlawi, S. M.; Nazir, W.; Iqbal, R.; Asghar, W.; Khalid, N. Formulation and Characterization of Oil-in-Water Nanoemulsions Stabilized by Crude Saponins Isolated from Onion Skin Waste. *RSC Adv.* **2020**, *10*, 39700–39707. https://doi.org/10.1039/d0ra07756a.

(7) Leibtag, S.; Peshkovsky, A. Cannabis Extract Nanoemulsions Produced by High-Intensity Ultrasound : Formulation Development and Scale-Up. *J. Drug Deliv. Sci. Technol.* **2020**, *60* (February), 101953. https://doi.org/10.1016/j.jddst.2020.101953.

(8) Jarzebski, M.; Siejak, P.; Smulek, W.; Fathordoobady, F.; Guo, Y.; Pawlicz, J.; Trzeciak, T.; Kowalczewski, P. L.; Kitts, D. D.; Singh, A.; Singh, A. P. Plant Extracts Containing Saponins Affects the Stability and Biological Activity of Hempseed Oil Emulsion System. *Molecules* **2020**, *25*, 1–16. https://doi.org/https://doi.org/10.3390/molecules25112696.

(9) Schreiner, T. B.; Santamaria-echart, A.; Ribeiro, A.; Peres, A. M.; Dias, M. M.; Pinho, S. P.; Barreiro, M. F. Formulation and Optimization of Nanoemulsions Using the Natural Surfactant Saponin from Quillaja Bark. *Molecules* **2020**, *25* (7), 1–14. https://doi.org/https://doi.org/10.3390/molecules25071538.

(10) Tsibranska, S.; Tcholakova, S.; Golemanov, K.; Denkov, N.; Arnaudov, L.; Pelan, E.; Stoyanov, S. D. Origin of the Extremely High Elasticity of Bulk Emulsions , Stabilized by Yucca Schidigera Saponins. *Food Chem.* **2020**, *316*, 126365.

(11) Bouhoute, M.; Taarji, N.; Vodo, S.; Kobayashi, I.; Zahar, M.; Isoda, H.; Nakajima, M.; Neves, M. A. Formation and Stability of Emulsions Using Crude Extracts as Natural Emulsifiers from Argan Shells. *Colloids Surfaces A Physicochem. Eng. Asp.* **2020**, *591*, 124536. https://doi.org/https://doi.org/10.1016/j.colsurfa.2020.124536.

(12) Ahmadi, O.; Jafarizadeh-Malmiri, H. Green Approach in Food Nanotechnology Based on Subcritical Water: Effects of Thyme Oil and Saponin on Characteristics of the Prepared Oil in Water Nanoemulsions. *Food Sci. Biotechnol.* **2020**. https://doi.org/https://doi.org/10.1007/s10068-019-00727-0.

(13) Gao, W.; Jiang, Z.; Du, X.; Zhang, F.; Liu, Y.; Bai, X.; Sun, G. Impact of Surfactants on Nanoemulsions Based on Fractionated Coconut Oil: Emulsification Stability and In Vitro Digestion. *J. Oleo Sci.* **2020**, *69* (3), 227–239.

(14) Wan, J.; Jin, Z.; Zhong, S.; Schwarz, P.; Chen, B.; Rao, J. Clove Oil-in-Water Nanoemulsion: Mitigates Growth of Fusarium Graminearum and Trichothecene Mycotoxin Production during the Malting of Fusarium Infected Barley. *Food Chem.* **2020**, *312*, 126120.

(15) Xu, X.; Sun, Q.; Mcclements, D. J. Enhancing the Formation and Stability of Emulsions Using Mixed Natural Emulsifiers: Hydrolyzed Rice Glutelin and Quillaja Saponin. *Food Hydrocoll.* **2019**, *89* (3), 396–405. https://doi.org/10.1016/j.foodhyd.2018.11.020.

(16) Kumari, S.; Choudhary, R. C.; Kumaraswamy, R. V; Bhagat, D.; Pal, A.; Raliya, R.; Biswas, P.; Saharan, V. Zinc-Functionalized Thymol Nanoemulsion for Promoting Soybean Yield. *Plant Physiol. Biochem.* **2019**, *145* (September), 64–74. https://doi.org/10.1016/j.plaphy.2019.10.022.

(17) Zhu, Z.; Wen, Y.; Yi, J.; Cao, Y.; Liu, F.; McClements, D. J. Comparison of Natural and Synthetic Surfactants at Forming and Stabilizing Nanoemulsions: Tea Saponin, Quillaja Saponin, and Tween 80. *J. Colloid Interface Sci.* **2019**, *536*, 80–87. https://doi.org/https://doi.org/10.1016/j.jcis.2018.10.024.

(18) Nejatian, M.; Abbasi, S. Formation of Concentrated Triglyceride Nanoemulsions and Nanogels: Natural Emulsifiers and High Power Ultrasound. *RSC Adv.* **2019**, *9* (49), 28330–28344.

(19) Wan, J.; Zhong, S.; Schwarz, P.; Chen, B.; Rao, J. Enhancement of Antifungal and Mycotoxin Inhibitory Activities of Food-Grade Thyme Oil Nanoemulsions with Natural Emulsifiers. *Food Control* **2019**, *106* (June), 106709.

(20) Riquelme, N.; Zúñiga, R. N.; Arancibia, C. Physical Stability of Nanoemulsions with Emulsifier Mixtures : Replacement of Tween 80 with Quillaja Saponin. *LWT - Food Sci. Technol.* **2019**, *111* (May), 760–766. https://doi.org/10.1016/j.lwt.2019.05.067.

(21) Sedaghat Doost, A.; Van Camp, J.; Dewettinck, K.; Van der Meeren, P. Production of Thymol Nanoemulsions Stabilized Using Quillaja Saponin as a Biosurfactant: Antioxidant Activity

Enhancement. *Food Chem.* **2019**, *293*, 134–143.
(22) Lv, S.; Zhang, Y.; Tan, H.; Zhang, R.; Mcclements, D. J. Vitamin E Encapsulation within Oil-in-Water Emulsions: Impact of Emulsifier Type on Physicochemical Stability and Bioaccessibility. *J. Agric. Food Chem.* **2019**, *67* (5), 1521–1529.
(23) Zheng, B.; Peng, S.; Zhang, X.; McClements, D. J. Impact of Delivery System Type on Curcumin Bioaccessibility: Comparison of Curcumin-Loaded Nanoemulsions with Commercial Curcumin Supplements. *J. Agric. Food Chem.* **2018**, *66* (41), 10816–10826.
(24) Lv, S.; Zhang, R.; Gu, J.; McClements, D. J.; Zhang, Y.; Tan, H. Vitamin E Encapsulation in Plant-Based Nanoemulsions Fabricated Using Dual-Channel Microfluidization: Formation, Stability, and Bioaccessibility. *J. Agric. Food Chem.* **2018**, *66* (40), 10532–10542. https://doi.org/10.1021/acs.jafc.8b03077.
(25) Weigel, F.; Weiss, J.; Decker, E. A.; McClements, D. J. Lutein-Enriched Emulsion-Based Delivery Systems: Influence of Emulsifiers and Antioxidants on Physical and Chemical Stability. *Food Chem.* **2018**, *242*, 395–403.
(26) Chen, Z.; Shu, G.; Taarji, N.; Barrow, C. J.; Nakajima, M.; Khalid, N.; Neves, M. A. Gypenosides as Natural Emulsifiers for Oil-in-Water Nanoemulsions Loaded with Astaxanthin: Insights of Formulation, Stability and Release Properties. *Food Chem.* **2018**, *261* (October 2017), 322–328.
(27) Kumari, S.; Kumaraswamy, R. V; Choudhary, R. C.; Sharma, S. S.; Pal, A.; Raliya, R.; Biswas, P.; Saharan, V. Thymol Nanoemulsion Exhibits Potential Antibacterial Activity against Bacterial Pustule Disease and Growth Promotory Effect on Soybean. *Sci. Rep.* **2018**, *8*, 1–12. https://doi.org/10.1038/s41598-018-24871-5.
(28) Sedaghat Doost, A.; Devlieghere, F.; Dirckx, A.; Van der Meeren, P. Fabrication of Origanum Compactum Essential Oil Nanoemulsions Stabilized Using Quillaja Saponin Biosurfactant. *J. Food Process. Preserv.* **2018**, No. 42, 1–12.
(29) Ryu, V.; Mcclements, D. J.; Corradini, M. G.; Yang, J. S.; Mclandsborough, L. Natural Antimicrobial Delivery Systems : Formulation , Antimicrobial Activity , and Mechanism of Action of Quillaja Saponin-Stabilized Carvacrol Nanoemulsions. *Food Hydrocoll.* **2018**, *82*, 442–450.
(30) Ralla, T.; Salminen, H.; Edelmann, M.; Dawid, C.; Hofmann, T.; Weiss, J. Oat Bran Extract (Avena Sativa L .) from Food by-Product Streams as New Natural Emulsifier. *Food Hydrocoll.* **2018**, *81*, 253–262. https://doi.org/https://doi.org/10.1016/j.foodhyd.2018.02.035.
(31) Reichert, C. L.; Salminen, H.; Badolato Bönisch, G.; Schäfer, C.; Weiss, J. Concentration Effect of Quillaja Saponin – Co-Surfactant Mixtures on Emulsifying Properties. *J. Colloid Interface Sci.* **2018**, *519*, 71–80.

(32) Chung, C.; Sher, A.; Rousset, P.; Julian, D. Impact of Oil Droplet Concentration on the Optical , Rheological , and Stability Characteristics of O / W Emulsions Stabilized with Plant-Based Surfactant : Potential Application as Non-Dairy Creamers. *Food Res. Int.* **2018**, *105*, 913–919.

(33) Taarji, N.; Rabelo da Silva, C. A.; Khalid, N.; Gadhi, C.; Hafidi, A.; Kobayashi, I.; Neves, M. A.; Isoda, H.; Nakajima, M. Formulation and Stabilization of Oil-in-Water Nanoemulsions Using a Saponins-Rich Extract from Argan Oil Press-Cake. *Food Chem.* **2018**, *246*, 457–463.

(34) Kadappan, A. S.; Guo, C.; Gumus, C. E.; Bessey, A.; Wood, R. J.; McClements, D. J.; Liu, Z. The Efficacy of Nanoemulsion-Based Delivery to Improve Vitamin D Absorption: Comparison of In Vitro and In Vivo Studies. *Mol. Nutr. Food Res.* **2018**, *62* (4), 1700836–1700844.

(35) Xu, F.; Pandya, J. K.; Chung, C.; McClements, D. J.; Kinchla, A. J. Emulsions as Delivery Systems for Gamma and Delta Tocotrienols: Formation, Properties and Simulated Gastrointestinal Fate. *Food Res. Int.* **2018**, *105*, 570–579.

(36) Ralla, T.; Herz, E.; Salminen, H.; Edelmann, M.; Dawid, C.; Hofmann, T.; Weiss, J. Emulsifying Properties of Natural Extracts from Panax Ginseng L. *Food Biophys.* **2017**, *12* (4), 479–490. https://doi.org/https://doi.org/10.1007/s11483-017-9504-5.

(37) Ralla, T.; Salminen, H.; Tuosto, J.; Weiss, J. Formation and Stability of Emulsions Stabilised by Yucca Saponin Extract. *Int. J. Food Sci. Technol.* **2017**, *53* (6), 1381–1388. https://doi.org/https://doi.org/10.1111/ijfs.13715.

(38) Luo, X.; Zhou, Y.; Bai, L.; Liu, F.; Deng, Y.; McClements, D. J. Fabrication of β-Carotene Nanoemulsion-Based Delivery Systems Using Dual-Channel Microfluidization: Physical and Chemical Stability. *J. Colloid Interface Sci.* **2017**, *490*, 328–335.

(39) Ralla, T.; Salminen, H.; Edelmann, M.; Dawid, C.; Hofmann, T.; Weiss, J. Sugar Beet Extract (Beta Vulgaris L.) as a New Natural Emulsifier: Emulsion Formation. *J. Agric. Food Chem.* **2017**, *65*, 4153–4160.

(40) Chung, C.; Sher, A.; Rousset, P.; Decker, E. A.; McClements, D. J. Formulation of Food Emulsions Using Natural Emulsifiers: Utilization of Quillaja Saponin and Soy Lecithin to Fabricate Liquid Coffee Whiteners. *J. Food Eng.* **2017**, *209*, 1–11.

(41) Luo, X.; Zhou, Y.; Bai, L.; Liu, F.; Zhang, R.; Zhang, Z.; Zheng, B.; Deng, Y.; McClements, D. J. Production of Highly Concentrated Oil-in-Water Emulsions Using Dual-Channel Micro Fluidization : Use of Individual and Mixed Natural Emulsifiers (Saponin and Lecithin). *Food Res. Int.* **2017**, *96*, 103–112.

(42) Faria, J. T. de; Oliveira, E. B. de; Minim, V. P. R.; Minim, L. A. Performance of Quillaja Bark Saponin and b -Lactoglobulin Mixtures on Emulsion Formation and Stability. *Food Hydrocoll.* **2017**, *67*, 178–188.

(43) Chung, C.; Sher, A.; Rousset, P.; Julian, D. Use of Natural Emulsifiers in Model Coffee Creamers : Physical Properties of Quillaja Saponin-Stabilized Emulsions. *Food Hydrocoll.* **2017**, *67*, 111–119.

(44) Tippel, J.; Gies, K.; Harbaum-piayda, B.; Steffen-heins, A.; Drusch, S. Composition of Quillaja Saponin Extract Affects Lipid Oxidation in Oil-in-Water Emulsions. *Food Chem.* **2017**, *221*, 386–394.

(45) Liu, F.; Zhu, Z.; Ma, C.; Luo, X.; Bai, L.; Decker, E. A.; Gao, Y.; Mcclements, D. J. Fabrication of Concentrated Fish Oil Emulsions Using Dual-Channel Microfluidization: Impact of Droplet Concentration on Physical Properties and Lipid Oxidation. *J. Agric. Food Chem.* **2016**, *64* (50), 9532–9541. https://doi.org/10.1021/acs.jafc.6b04413.

(46) Kaur, K.; Kumar, R.; Mehta, S. K. Formulation of Saponin Stabilized Nanoemulsion by Ultrasonic Method and Its Role to Protect the Degradation of Quercitin from UV Light. *Ultrason. Sonochem.* **2016**, *31*, 29–38.

(47) Choudhry, Q. N.; Kim, M. J.; Kim, T. G.; Pan, J. H.; Kim, J. H.; Park, S. J.; Lee, J. H.; Kim, Y. J. Saponin-Based Nanoemulsification Improves the Antioxidant Properties of Vitamin A and E in AML-12 Cells. *Int. J. Mol. Sci.* **2016**, *17* (1406).

(48) Bai, L.; Huan, S.; Gu, J.; McClements, D. J. Fabrication of Oil-in-Water Nanoemulsions by Dual-Channel Microfluidization Using Natural Emulsifiers: Saponins, Phospholipids, Proteins, and Polysaccharides. *Food Hydrocoll.* **2016**, *61*, 703–711.

(49) Parthasarathi, S.; Muthukumar, S. P.; Anandharamakrishnan, C. The Influence of Droplet Size on the Stability, in Vivo Digestion, and Oral Bioavailability of Vitamin E Emulsions. *Food Funct.* **2016**, *7*, 2294–2302.

(50) Rosa, M. T. M. G.; Silva, E. K.; Santos, D. T.; Petenate, A. J.; Meireles, M. A. A. Obtaining Annatto Seed Oil Miniemulsions by Ultrasonication Using Aqueous Extract from Brazilian Ginseng Roots as a Biosurfactant. *J. Food Eng.* **2016**, *168*, 68–78.

(51) Zhang, J.; Bing, L.; Reineccius, G. A. Comparison of Modified Starch and Quillaja Saponins in the Formation and Stabilization of Flavor Nanoemulsions. *Food Chem.* **2016**, *192*, 53–59.

(52) Uluata, S.; McClements, D. J.; Decker, E. A. Physical Stability, Autoxidation, and Photosensitized Oxidation of ω-3 Oils in Nanoemulsions Prepared with Natural and Synthetic Surfactants. *J. Agric. Food Chem.* **2015**, *63* (42), 9333–9340. https://doi.org/https://doi.org/10.1021/acs.jafc.5b03572.

(53) Zhang, J.; Bing, L.; Reineccius, G. A. Formation, Optical Property and Stability of Orange Oil Nanoemulsions Stabilized by Quillaja Saponins. *LWT - Food Sci. Technol.* **2015**, *64* (2), 1063–1070.

(54) Ozturk, B.; Argin, S.; Ozilgen, M.; McClements, D. J. Nanoemulsion Delivery Systems for Oil-Soluble Vitamins : Influence of Carrier Oil Type on Lipid Digestion and Vitamin D3 Bioaccessibility. *FOOD Chem.* **2015**, *187*, 499–506. https://doi.org/10.1016/j.foodchem.2015.04.065.

(55) Ozturk, B.; Argin, S.; Ozilgen, M.; McClements, D. J. Formation and Stabilization of Nanoemulsion-Based Vitamin E Delivery Systems Using Natural Surfactants: Quillaja Saponin and Lecithin. *J. Food Eng.* **2014**, *142*, 57–63. https://doi.org/https://doi.org/10.1016/j.jfoodeng.2014.06.015.

(56) Yang, Y.; Leser, M. E.; Sher, A. A.; McClements, D. J. Formation and Stability of Emulsions Using a Natural Small Molecule Surfactant: Quillaja Saponin (Q-Naturale®). *Food Hydrocoll.* **2013**, *30* (2), 589–596. https://doi.org/https://doi.org/10.1016/j.foodhyd.2012.08.008.

(57) Mayer, S.; Weiss, J.; McClements, D. J. Vitamin E-Enriched Nanoemulsions Formed by Emulsion Phase Inversion : Factors Influencing Droplet Size and Stability. *J. Colloid Interface Sci.* **2013**, *402*, 122–130.
